# Comparing different motion correction approaches for resting-state functional connectivity analysis with functional near-infrared spectroscopy data

**DOI:** 10.1117/1.NPh.11.4.045001

**Published:** 2024-10-03

**Authors:** Costanza Iester, Laura Bonzano, Monica Biggio, Simone Cutini, Marco Bove, Sabrina Brigadoi

**Affiliations:** a University of Genoa, Department of Neuroscience, Rehabilitation, Ophthalmology, Genetics, Maternal and Child Health, Genoa, Italy; b IRCCS Ospedale Policlinico San Martino, Genoa, Italy; c University of Padua, Department of Developmental Psychology and Socialization, Padua, Italy; d University of Genoa, Department of Experimental Medicine, Section of Human Physiology, Genoa, Italy

**Keywords:** resting-state functional connectivity, functional near-infrared spectroscopy, motion artifacts, motion detection, motion correction

## Abstract

**Significance:**

Motion artifacts are a notorious challenge in the functional near-infrared spectroscopy (fNIRS) field. However, little is known about how to deal with them in resting-state data.

**Aim:**

We assessed the impact of motion artifact correction approaches on assessing functional connectivity, using semi-simulated datasets with different percentages and types of motion artifact contamination.

**Approach:**

Thirty-five healthy adults underwent a 15-min resting-state acquisition. Semi-simulated datasets were generated by adding spike-like and/or baseline-shift motion artifacts to the real dataset. Fifteen pipelines, employing various correction approaches, were applied to each dataset, and the group correlation matrix was computed. Three metrics were used to test the performance of each approach.

**Results:**

When motion artifact contamination was low, various correction approaches were effective. However, with increased contamination, only a few pipelines were reliable. For datasets mostly free of baseline-shift artifacts, discarding contaminated frames after pre-processing was optimal. Conversely, when both spike and baseline-shift artifacts were present, discarding contaminated frames before pre-processing yielded the best results.

**Conclusions:**

This study emphasizes the need for customized motion correction approaches as the effectiveness varies with the specific type and amount of motion artifacts present.

## 1 Introduction

Resting-state functional connectivity (RSFC) is largely studied in the field of neuroscience. RSFC investigates the correlation of slow signal changes (<0.1  Hz) among different brain areas in the absence of any stimulus or task.^1^ The correlation structure of spontaneous activity in RSFC maps can provide insight into the intrinsic functional architecture of the human brain. Moreover, RSFC has been found altered in some pathological conditions (e.g., stroke, autism, and multiple sclerosis) and could thus be considered a potential biomarker of a healthy brain.^2^^–^^5^

Biswal and colleagues^6^ first reported the correlation between the left and right somatosensory motor cortices at rest using functional magnetic resonance imaging (fMRI). Afterward, other resting-state networks were discovered (e.g., visual, attention, and default mode networks) using fMRI.^7^^–^^9^ Although fMRI could be considered the gold standard technique to study RSFC, given its high spatial and depth resolution, there are some populations (e.g., infants or particular clinical populations) who can be hardly acquired in the MRI scanner.

A promising alternative to fMRI for studying RSFC is functional near-infrared spectroscopy (fNIRS). fNIRS monitors non-invasively cortical hemodynamic variations using red and near-infrared light. fNIRS has been shown to be reproducible,^10^ feasible,^11^ and reliable^12^^,^^13^ in characterizing functional connectivity at rest. Compared with fMRI, it is more participant friendly, i.e., it is quieter; has more tolerance to motion artifacts (MAs); and allows participants to sit or stand instead of lying down in the MRI scanner, thus resulting in more ecological acquisitions. fNIRS can be used in any population (from newborns to the elderly, both healthy or pathological) and can be easily applied multiple times to study RSFC replicability, given its lower cost compared with fMRI. Importantly, typical fNIRS systems have a sampling rate of ∼10  Hz, which decreases the risk of aliasing between high [e.g., heart rate (∼1  Hz)] and low frequencies (<0.1  Hz) compared with fMRI.^1^ There are some limitations when using fNIRS instead of fMRI. First, with fNIRS, only cortical activity can be mapped, and no information on deeper brain areas can be measured. Second, most fNIRS systems do not have a high enough number of sources and detectors to cover the whole head, thus further limiting the brain areas for which RSFC could be computed. Recent advances in hardware development, however, have demonstrated the feasibility to reach high-density coverage of the whole cortex in the near future, thus increasing the spatial resolution of the RSFC mapping.^14^^,^^15^

The standard pre-processing steps for fNIRS signals involve removing noisy channels, correcting for motion artifacts and physiological noise contamination, and band-pass filtering. The most challenging and important steps are physiological noise regression and motion correction.

Several studies have demonstrated the importance of correcting for physiological noise contamination, which could bias the results.^16^^–^^18^ Physiological noise contamination is mostly due to blood pressure, respiration, and heartbeat changes, as well as the strong contribution in fNIRS signals of extra-cerebral (skin and skull) layers, which are highly vascularized. Extra-cerebral physiological noise contribution is usually reduced by regressing, from standard channel signals, the signals acquired from short-separation channels,^19^^,^^20^ which preponderantly measure the extra-cerebral contributions. In the resting-state field, it has been shown that systemic physiology can overestimate RSFC.^21^ Recently, Abdalmalak et al.^22^ used the regression of short-separation channels to overcome this problem of systemic physiology, obtaining reduced inter-subject and intra-subject variability. Lanka and colleagues^23^ further investigated this problem in a fully simulated scenario by testing different analysis pipelines and evaluating their sensitivity and specificity in estimating statistically valid connectivity matrices. Their results suggested that incorporating short-separation channels in partial correlation models reduced spurious correlations due to synchronous physiological fluctuations. Furthermore, they also demonstrated that robust statistical methods and pre-whitening seem to be effective in mitigating motion artifacts and autocorrelation.

Although several studies have demonstrated the importance of correcting motion artifacts in task-based fNIRS acquisitions and provided guidelines for choosing the best motion correction technique for the available dataset,^24^^–^^28^ very little has been done to tackle this problem in RSFC studies. Resting-state acquisitions do not involve participants’ movements, as several task-based experiments do; nevertheless, resting-state acquisitions are not immune from motion artifacts. Motion artifacts are mainly caused by a decoupling between the skin and the optode, which can cause a sudden change in the measured light intensity. Optode-skin decoupling can be caused by facial movements, particularly movements involving the jaw, but also by a simple eyebrow-raising.^29^ Motion artifacts can be classified into spikes, baseline shifts, and low-frequency variations. Motion artifact categories differ in amplitude and frequency content of the artifact. Spikes generally have high frequency and amplitude and are more easily detected than low-frequency variations. Baseline shifts cause a variation in the signal baseline and are mostly due to changes in the detected light intensity due to the optode-skin decoupling.

To the best of our knowledge, the first and only paper trying to tackle the motion artifact issue in resting-state fNIRS was the one published in 2015 by Selb and colleagues.^30^ They studied the effect of MAs on resting-state data using the interhemispheric correlation (IHC) (i.e., the correlation coefficient between symmetrical time series of oxyhemoglobin oscillations) as a metric of interest. Stroke patients and healthy adults were recruited for the study, and they were asked to stay still for 10 min in a supine position. First, the authors computed the IHC using the whole acquired data, which contained motion artifacts, and they found a significantly higher IHC in healthy adults than in patients. However, the healthy group dataset had less MAs than the patient one. Therefore, the authors increased the number of MAs in the heathy adult dataset to achieve a similar amount of artifact in the two groups, with the aim to understand whether the presence of MAs could have biased the comparison. MAs were extracted from the patient dataset and then added to the healthy adult one. Comparing the patient dataset with the newly created healthy adult dataset, no significant differences were found, revealing an important impact of MAs on IHC computation. The authors further investigated the impact of MAs on IHC by pre-processing the healthy adult dataset with high contamination of MAs with several pipelines, which differed only for the motion correction step. Their results revealed that the best motion correction approach was to discard segments of data contaminated by MAs.

A limitation of this study was that they investigated only the impact of motion artifacts on the correlation between symmetric channels, one on the right and one on the left hemisphere. Furthermore, short-separation channel regression was not performed, thus increasing the chance that the data could be highly contaminated by physiological noise, which could further bias the results.

Despite the insights of this article, in the following years, researchers publishing papers on RSFC used several different approaches to deal with motion artifacts. Some papers followed the suggestion of Selb and colleagues and discarded segments of signals contaminated by motion artifacts.^31^^,^^32^ Several others decided to opt for a no motion correction approach.^14^^,^^15^^,^^33^^–^^36^ Another subset of papers applied various motion correction techniques to reduce motion artifacts before computing RSFC, overlooking the impact and possible bias of the correction (spline interpolation technique;^37^^–^^41^ wavelet filtering technique^42^^–^^45^).

Spline interpolation is a technique widely used to correct motion artifacts in task-based studies.^46^ Its great advantage is that it extracts and corrects only the segments of data identified as MAs, leaving the remaining time series untouched. The spline interpolation technique is simple and fast; however, it requires a reliable technique to pre-identify MAs and may leave some residual high-frequency noise after the correction. Wavelet filtering^47^ is another widely employed technique for MA correction. It does not require a previous step to identify motion artifacts, and it is ideal for removing high-frequency content; however, it is computationally expensive and modifies the entire time series, not only the contaminated segments.

A few RSFC studies have applied the combination of spline and wavelet,^22^^,^^48^ under the hypothesis that this combination should work better when several motion artifacts of different types (e.g., spikes, baseline shifts) are present, as demonstrated in task-based activation studies.^25^^,^^49^ Other researchers have employed a combination of the temporal derivative distribution repair (TDDR)^50^ method followed by the wavelet filtering one.^51^

This high variability in the choice of the motion correction step in RSFC pipelines and the scarcity of comparison papers demonstrating the impact of motion correction techniques on the connectivity results suggests the need for user guidelines on how to best correct motion artifacts in resting-state data. Importantly, these guidelines are probably different from the one for task-based data and could be modulated by the degree of motion artifact contamination in the data, as demonstrated for task-based studies by Di Lorenzo et al.^25^

One important requirement of some motion correction techniques [i.e., spline interpolation and target principal component analysis (tPCA)^52^] is the pre-identification of the motion artifacts in the signals. The performance of the technique relies on the optimal identification of motion artifacts. In the literature, the most common approach for identifying motion artifacts is the one developed in Homer2/Homer3,^53^ which identifies as motion artifacts the segments of data around time points that exhibit a signal change greater than a standard deviation or an amplitude threshold (see Sec. 2.2.1). The performance of this approach might depend on the degree of motion artifact contamination in the signal. When the signal is highly contaminated, as might be in newborn acquisition, the method might fail in accurately detecting motion artifacts. Recently, Yang and colleagues^54^ proposed a new motion detection approach. Whereas Homer computed the standard deviation threshold considering the standard deviation of the entire signal, Yang and colleagues considered the standard deviation of the noise-free portion of the signal only. They demonstrated that their approach outperformed the Homer one when the dataset is highly contaminated by MAs. The comparison was performed on a newborn dataset that contained a fixed percentage of MAs in the range of 25% to 35%. Moreover, Sherafati and colleagues^55^ proposed a new artifact identification technique to deal with the challenge of motion-induced artifacts in both task-based and RSFC high-density diffuse optical tomography (HD-DOT): the global variance of temporal derivatives (GVTD). GVTD evaluates motion by analyzing spatial patterns across measurement channels and demonstrates a strong correlation with external measures of motion. It exhibits high sensitivity and specificity in detecting instructed motion. When applied to the RSFC HD-DOT data, GVTD-based motion correction improves spatial similarity to fMRI mapping, showing a better performance than other commonly used fNIRS motion correction methods, such as TDDR, wavelet filtering, and tPCA. What is missing in the literature is a comparison of the three approaches dependent on the degree of motion artifact contamination.

The aim of this paper is to evaluate the impact of discarding, not correcting, or correcting motion artifacts on RSFC as a function of the degree and the type of motion artifact contamination in the signals and provide guidelines to users for this important pre-processing step. We acquired resting-state data from a population of compliant healthy adults, which resulted in a dataset with less than 5% contamination by motion artifacts. We extracted the available motion artifacts from this dataset, simulated types of motion artifacts not present in the dataset (e.g., baseline shifts), and created a Motion Artifact Database. Several new datasets with different contaminations and typologies of motion artifacts were then created by randomly extracting MAs from the database and adding them to the original dataset. First, we compared for each dataset the three different motion identification approaches to evaluate their performance as a function of the amount of motion artifact contamination. Second, we tested several pre-processing pipelines using different approaches for motion correction (discard, no motion correction, spline, spline Savitzy-Golay, wavelet, a combination of spline and wavelet, TDDR, and tPCA). Differences between the functional connectivity, measured with the correlation matrix and obtained after motion correction, and the functional connectivity considered to be the ground truth (the discard pipeline) were estimated to evaluate the performance of each pipeline as a function of motion artifact contamination.

## 2 Methods

**Fig. 1:**
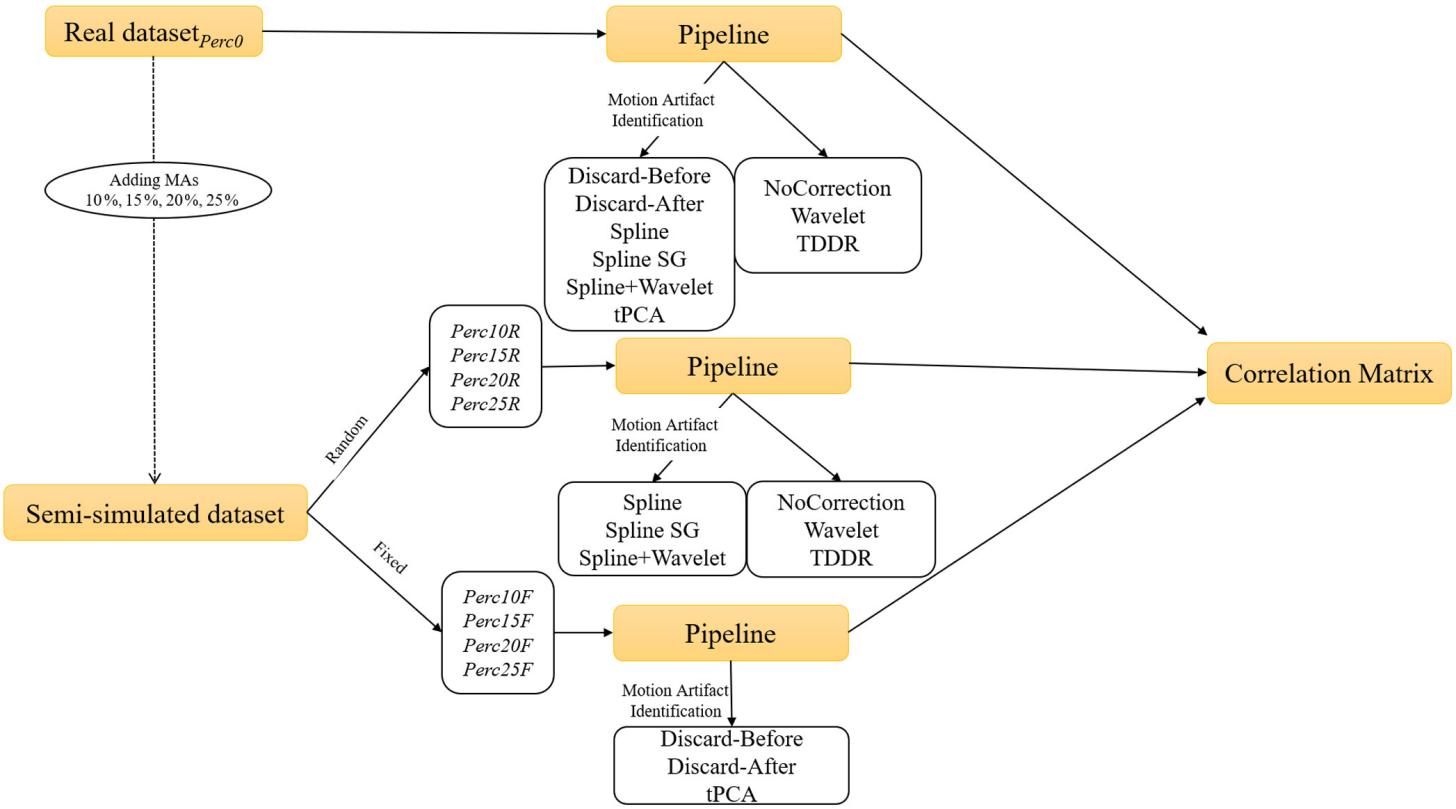
Block diagram of the analysis procedure. Several correlation matrixes were computed on different datasets and after specific pipelines.

Figure 1 summarizes the steps undertaken to create the semi-simulated scenario and to pre-process the data with different pipelines before computing the correlation matrixes used in the comparison.

### 2.1 Datasets

#### 2.1.1 Real dataset

Thirty-five healthy adults (mean age = 32.5 years, SD = 13.7, 22 females, age range [21, 74] years) participated in the experiment after providing written informed consent. The work was carried out in accordance with the Code of Ethics of the World Medical Association (Declaration of Helsinki). The study was approved by the Regional Ethics Committee of Azienda Ospedaliera “San Martino,” Genoa, Italy (P.R. 271REG2017).

The experiment consisted of 15 min of resting state. Participants were seated in a dimly lit room, and they were asked to keep their eyes closed and remain as still as possible for the entire duration of the resting-state acquisition.

**Fig. 2:**
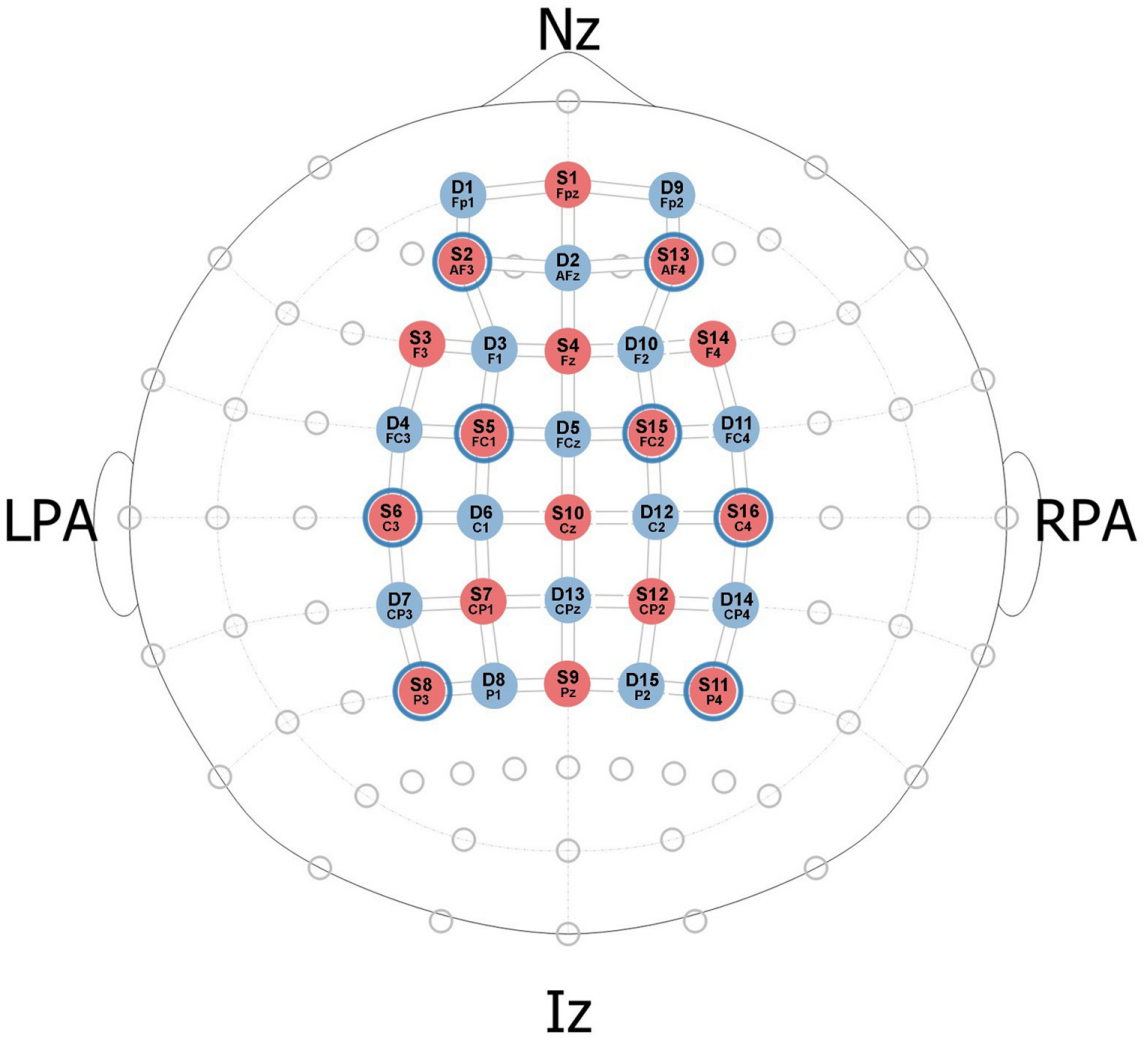
Probe layout and locations of the optodes (positions in the 10–10 EEG system) and channels. Red dots represent sources, blue dots indicate detectors, and lines represent channels.

The fNIRS data was acquired with the NIRSport2 system (NIRSport, NIRx Medical Technologies, Berlin, Germany) equipped with 16 sources (at 760 and 850 nm) and 16 detectors. Optodes were arranged in 50 standard channels with a source-detector distance of 3 cm and eight short-separation (SS) channels with a source-detector distance of 8 mm, covering frontal, parietal, premotor, motor, and sensory brain areas (see Fig. 2). The sampling frequency was set at 8.7 Hz.

The real dataset acquired during this resting-state condition is defined in the following as the *Perc0* dataset, as for all participants, the percentage of motion artifacts in each channel was <5% of the total acquisition time.

#### 2.1.2 Semi-simulated datasets

To create new semi-simulated datasets with different percentages of MAs, MAs from “Perc0” dataset were extracted, and a Motion Artifact Database was created. All steps were performed in MATLAB (R2021b, MathWorks, Natick, Massachusetts, United States). MAs were identified by applying the Homer3 function *hmrR_MotionArtifactByChannel* on changes in optical density data. This function detects the signal exceeding a threshold in a change of amplitude (AMPthresh) or/and a threshold in a change of standard deviation (STDEVthresh) within a predefined time-window (tMotion) and marks as artifacts the data points around the detected motion (± tMask). Here, we used AMPthresh = 0.5; STDEVthresh = 0.5; tMotion = 0.5; and tMask = 1. MAs were defined and identified as artifactual parts of the signal with at least four samples of good signal preceding and following the artifact. Each detected MA was saved in the Motion Artifact Database after removing its mean. Furthermore, each artifact was coupled in the database with another parameter called Alpha, which characterizes the channel from where the MA came and keeps track of the amplitude range of the original signal. The Alpha parameter was computed on the signal free of MAs with a sliding window approach (2 min, overlapping) and taking the median of the amplitude of all sliding windows. The amplitude was computed as the difference between the maximum and minimum signal values in the sliding window. Both the average-free MA and its Alpha parameter were required to properly add scaled motion artifacts in the semi-simulated datasets.

In the original dataset, spike-type motion artifacts were predominantly detected. As a result, multiple datasets were generated: each contained a different percentage of exclusively spike-type MAs (Only Spikes datasets). Because another type of motion artifact common in fNIRS acquisition and hard to deal with is baseline shifts, additional datasets were created to encompass both simulated baseline shifts and real spikes MAs (BS + Spike datasets). This approach aimed to provide insights into the optimal pipeline, considering not only the quantity but also the specific types of MAs present.

**Fig. 3:**
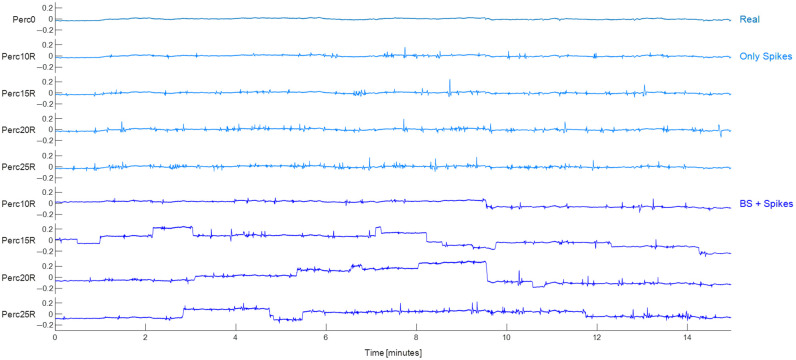
Representative channel for each dataset for the dataset built adding MAs randomly in each channel (random dataset). *Perc0* is displayed at the top of the figure, followed by the semi-simulated datasets at 10%, 15%, 20%, and 25%, for both the Only Spikes and BS+Spikes datasets.

Eight semi-simulated Only Spikes datasets were created adding MAs from the Motion Artifact Database to changes in optical density data of the *Perc0* dataset. Datasets differed for the percentage of added MAs over the total duration (10%, 15%, 20%, or 25% of the total duration time) and for the position of MAs (in random R or fixed F positions among channels of the same participant): *Perc10R*, *Perc15R*, *Perc20R*, *Perc25R*, *Perc10F*, *Perc15F*, *Perc20F*, and *Perc25F*. For each channel of the *Perc0* dataset, the Alpha parameter was computed as previously done when creating the Motion Artifact Database. The ratio between the Alpha of the selected channel and the Alpha of the database channel resulted in the scale parameter. Before adding each MA in the channel, the artifact was multiplied by the scale parameter. For each channel, MAs were randomly extracted from the Motion Artifact Database until the imposed percentage of MAs was achieved. Then, the extracted MAs were either inserted in randomly selected frames in each channel (random dataset, see Fig. 3) or they were randomly inserted only in the first channel of each participant, whereas in all other channels of that participant, MAs were inserted in the same temporal frames of the first channel, to mimic actual movement artifacts (fixed dataset, see Fig. S1 in the Supplementary Material). In both dataset types (random and fixed), possible overlapping of MAs was prevented.

An additional eight semi-simulated datasets were generated by introducing both baseline shifts and spike motion artifacts (MAs) into the signal. Specifically, baseline shifts were simulated by applying modifications to the optical intensity data of the *Perc0* dataset, and spikes extracted from the Motion Artifact Database were incorporated into changes in optical density data obtained after introducing baseline shifts. Similar to the Only Spikes datasets, the BS+Spikes datasets varied based on the percentage of added MAs over the total duration and the position of MAs: *Perc10R*, *Perc15R*, *Perc20R*, *Perc25R*, *Perc10F*, *Perc15F*, *Perc20F*, and *Perc25F*. First, baseline shifts were introduced in the intensity data. The proportion of added baseline shifts ranged from 0% to 10% of the total MA percentage within the dataset. Baseline shifts led to alterations in the signal baseline, achieved by adding or subtracting a constant value. This manipulation involved modifying the baseline signal by increasing or decreasing it by 5% to 15% (random selection) of the original baseline. The transition of the signal was gradual rather than instantaneous, spanning a few seconds (from 0.6 to 1.7 s, random selection) corresponding to the length of the motion artifact. The sum of all BS lengths was adjusted to account for 0% to 10% of the total artifacts in that dataset. This length value was saved to determine the subsequent requirement of spike-type artifacts. Notably, in each dataset, the sum of the two types of MAs (BS and Spikes) equaled the total specified percentage. When introducing BS MAs, two essential constraints were observed: optical intensity data could not become negative and the overall signal change should be smaller than 0.4 (intensity value). These constraints were essential to accurately replicating real-world conditions. Following the incorporation of baseline shift artifacts, optical intensity values were converted into optical density, and the same steps elucidated in the Only Spikes datasets were executed to introduce spike artifacts to this dataset (see Fig. 3).

### 2.2 Motion Identification Techniques

In the semi-simulated datasets, the position of MAs is known, providing a ground truth to evaluate the performance of motion identification techniques, which is essential for motion correction techniques relying on previous MA identification. We compared the performance of three different approaches: (a) the standard and widely employed identification approach implemented in Homer2 and Homer3,^53^ (b) a newly proposed approach,^54^ and (c) the GVTD approach.^55^

#### 2.2.1 Fixed standard deviation identification approach

The motion artifact detection technique implemented in Homer2/3 identifies as a motion artifact a segment of data around a time point (tMask) that exhibits a signal change greater than a standard deviation threshold (STDthresh times the standard deviation of the entire signal) or greater than an amplitude threshold (AMPthresh) within a time window of length tMotion. These thresholds are subjective and dataset-dependent and are usually chosen with a trial-and-error approach based on visual inspection. After visually inspecting our data using varying STDthresh (STDthresh = 7 to 15), we selected STDthresh = 12 as the best compromise for this dataset, with AMPthresh = 0.5, tMotion = 0.5, and tMask = 1. We used STDthresh values within the range of 7 to 15 because these are the values that are predominantly and most frequently used in the literature.^25^^,^^30^^,^^54^ Starting from this range, we identified the best one for our data, as standard practice in the fNIRS field.

#### 2.2.2 Adaptable standard deviation identification approach

The approach developed by Yang and colleagues^54^ is based on the Homer2/3 approach, but the computation of the threshold on the standard deviation is different. This method aims to slightly overcome the subjectivity of the Homer2/3 approach and to overcome its poor performance when the dataset is severely contaminated by motion artifacts. In Yang’s approach, the AMPthresh is computed and used as in Homer2/3, whereas the threshold for the standard deviation is computed only on the noise-free physiology of the signal. The entire signal is divided into small segments of 4 s as suggested in Yang’s paper.^54^ Hypothetically, segments could include both noiseless data and data with artifacts. For each segment, the standard deviation is computed and then all obtained values are sorted (from the smallest to the highest). Under the hypothesis that motion artifacts should induce higher standard deviations, to compute the actual standard deviation of the motion-free segments, only the standard deviations of the first 30% of segments (the ones with the smallest standard deviations) are averaged to obtain a measure of the standard deviation of the signal.^54^ Therefore, the standard deviation was calculated only from the physiological oscillations of motion-free segments. The new standard deviation value was multiplied by a fixed STDthresh = 7. AMPthresh and tMotion were set as for the Homer2/3 approach, and tMask was set to 0.5. Figure S2 in the Supplementary Material reports an example of MA identification using the adaptable standard deviation identification approach. The code is available on GitHub (https://github.com/sbrigadoi/motionDetection).

#### 2.2.3 Global variance of temporal derivatives approach

The GVTD^55^ serves as an indicator of the overall instantaneous alterations within optical temporal profiles. At each time point, GVTD is determined by calculating the root mean square (RMS) of the temporal derivatives across a collection of measurements (i.e., channels) or voxels. A straightforward analytical formula for computing GVTD is

$$g=[\begin{array}{c} g_{1}\\ .\\ .\\ g_{M} \end{array}],\;g_{i}=\sqrt{\frac{1}{N}\overset{N}{\underset{j=1}{\sum }}(y_{ji}-y_{ji-1}{)}^{2}},\;g_{i}\in R>0.$$

In the equation, g represents the GVTD vector, where $y_{ji}\in R$ denotes either the change in optical density or the change in molar concentrations of HbO or HbR at channel j. The index i refers to the time points, N represents the total number of channels, and M indicates the number of time points. To censor data using the GVTD time-course, good data were separated from motion artifacts using an outlier detection strategy. A noise threshold was computed considering both the GVTD distribution mode and *nStd* times the standard deviation on the left side of the mode. The right tail of the GVTD distribution corresponds to motion artifacts. The value of *nStd* controls the trade-off between the exclusion of artifacts versus data loss. Here, we set the *nStd* value to 3 as suggested by Sherafati and colleagues ^55^

#### 2.2.4 Evaluation of performance for motion identification techniques

To evaluate and compare the performance of the three identification techniques, two metrics were employed. First, the percentage of MAs detected in each channel by the three techniques was compared with the percentage of MAs that had been added to that channel (ground truth). The percentage of MAs detected was computed as the percent ratio of the number of frames identified as MAs to the total number of frames. This metric, however, does not provide any information on the accuracy of the detection, i.e., whether the detected MAs are exactly in the temporal positions where they had been added. The second metric employed yields this information by performing a sensitivity analysis. Comparing ground truth MA positions and identified MA positions, it was possible to classify temporal frames as MAs correctly identified (true positive - TP) and no-MAs correctly not identified (true negative - TN). The accuracy metric is computed as

$$\text{Accuracy}\;(\%)=\frac{\mathrm{TP}+\mathrm{TN}}{\mathrm{Ntot}}x\;100\%,$$ (1)

where Ntot corresponds to the total number of frames. The higher the accuracy is, the better the performance is.

### 2.3 Motion Correction Techniques

The performance of spline interpolation, spline and Savitzky-Golay filtering, wavelet filtering, the combination of spline and wavelet, TDDR, and tPCA was evaluated on real and semi-simulated datasets. Wavelet filtering and the combination of spline interpolation and wavelet filtering were tested using different *iqr* parameters to evaluate its impact on the connectivity results. Two other approaches were used in the comparison: no motion correction (not performing any correction of the data) and discard, based on the removal of the segments of data identified as motion artifacts from the signal. Two distinct methods for discarding artifacts were evaluated. The first approach, commonly encountered in existing literature,^31^^,^^56^^,^^57^ involved identifying motion artifacts and segregating motion-free segments. Subsequently, motion-free segments exceeding a duration of 20 s were individually analyzed and combined at the end of the pre-processing, before computing the correlation (discard-before approach). By contrast, the second method involved identifying motion artifacts but analyzing the whole signal, leaving the artifacts inside. At the end of the pre-processing, before computing the correlation matrix, frames recognized as artifacts were removed (discard-after approach).

Given the best performance of the adaptable standard deviation identification approach (see Sec. 3.1), this latter approach was used before all motion correction techniques relying on previous MA identification.

#### 2.3.1 Spline interpolation

The spline interpolation method was proposed by Scholkmann et al.^46^ This method models motion artifacts via cubic spline interpolation; the resulting spline interpolation is then subtracted from the original signal. Because this subtraction creates differences in the signal levels, every MA segment should be shifted to ensure a continuous signal. The spline interpolation method corrects only previously detected motion artifacts and therefore relies on motion identification techniques. The degree of the spline function is determined by the spline interpolation parameter (p-Spline). This parameter is designed so that, when it is equal to zero, the motion artifact is represented with a least-squares straight-line fit. On the other hand, when it is equal to one, the motion artifact is modeled using a natural cubic spline interpolation. Therefore, utilizing motion correction with p_Spline = 0 only removes the straight-line component of the artifact, whereas a model with p_Spline = 1 closely approximates the artifact, resulting in a nearly constant residual signal after subtraction. Here, the parameter was set to 0.99 as in the previous work by Scholkmann et al.^46^

#### 2.3.2 Spline interpolation and Savitzky-Golay filtering

Jahani et al.^58^ introduced a novel approach that combines spline interpolation with Savitzky-Golay (SG) filtering to address various types of motion artifacts, with the idea of correcting the residual high-frequency noise that is usually left after the spline interpolation approach. Spline interpolation is effective for correcting baseline shifts, whereas SG filtering is suitable for correcting high-frequency spikes. The authors recommended using this combined approach when the signal-to-noise ratio (SNR) of the data is greater than 3. However, they suggested employing only the SG filtering part of the algorithm when the SNR<3. The algorithm works first by correcting baseline shifts with the spline interpolation (see Sec. 2.3.1) and then smoothing out the remaining spikes using the Savitzky-Golay filter, which is a digital filter designed for smoothing data. This filter replaces each data point in the signal series with a new value determined by fitting a cubic curve to a subset of neighboring data points. The size of this subset is determined by the parameter FrameSize_sec. Here, the parameter was set to 10 s.

#### 2.3.3 Wavelet filtering

The wavelet-based motion artifact removal method was proposed by Molavi and Dumont.^47^ It decomposes the time-course of the signal into the wavelet domain using the general discrete wavelet transformation. The model assumes that the measured signal is a linear combination of the physiological signal of interest and the artifacts, the wavelet coefficients have a Gaussian probability distribution, and the hemodynamic response is smoother and slower than motion artifacts. Thus, coefficients accounting for the evoked response are centered around zero, whereas the outliers of the Gaussian distribution are the coefficients accounting for the motion artifacts. Therefore, to remove motion artifacts in the temporal time series, outlying coefficients are set to zero before reconstructing the signal with the inverse discrete wavelet transform. The threshold to define outliers is computed by multiplying the inter-quartile range by a tuning parameter *iqr* and adding/subtracting the obtained value to the third/first quartile. In this study, *iqr* was set to either 1.5, 1.2, 0.8, or 0.5, for a total of four wavelet approaches (wavelet 15, wavelet 12, wavelet 08, and wavelet 05). Concerning the choice of the *iqr* range tested, an *iqr* of 1.5 is the default value used in Homer, whereas 0.8 and 0.5 are values employed in various methodological works found in the literature.^25^^,^^30^ Finally, we introduced 1.2 to fill the gap between 0.8 and 1.5. The higher the *iqr* is, the fewer coefficients are deleted.

#### 2.3.4 Temporal derivative distribution repair

The TDDR approach works on the derivative of activity (fluctuations) assuming that, if motion artifacts are absent, fluctuations are normally distributed; most fluctuations are free of MAs, and MA fluctuations have a greater magnitude than non-motion fluctuation. This means that the MA contribution is large and infrequent. To delete the contribution of MA fluctuations, the TDDR method reduces the weights of abnormally large fluctuations through a robust regression. This method is advantageous because it does not require user-supplied parameters and performs well both in removing spike and baseline shift artifacts.

#### 2.3.5 Targeted principle component analysis

The targeted principal component analysis (tPCA) method was proposed by Yücel et al.^52^ tPCA applies the PCA techniques only on the frames identified as motion artifacts, therefore relying on an MA identification technique. The spline interpolation technique works on a channel-by-channel basis, whereas the tPCA technique is a multi-channel approach. Therefore, a time point is identified as MA for all channels if it is identified as MA in at least one channel. All segments identified as MAs from all channels are merged in a matrix and submitted to PCA. The principal components are then ranked in decreasing order of explained percent variance. The first N components that allow for removing up to 97% of the variance in the data are removed before reassembling the data. These corrected segments are then inserted back into the original time-series data with the same shifting procedure of the spline interpolation method. This procedure is repeated multiple times, re-identifying any residual motion artifacts, and stopped either when no more motion artifacts are identified or when reaching the maximum number of allowed iterations (maxIter = 5).

### 2.4 Data Pre-processing and Analysis

**Fig. 4:**
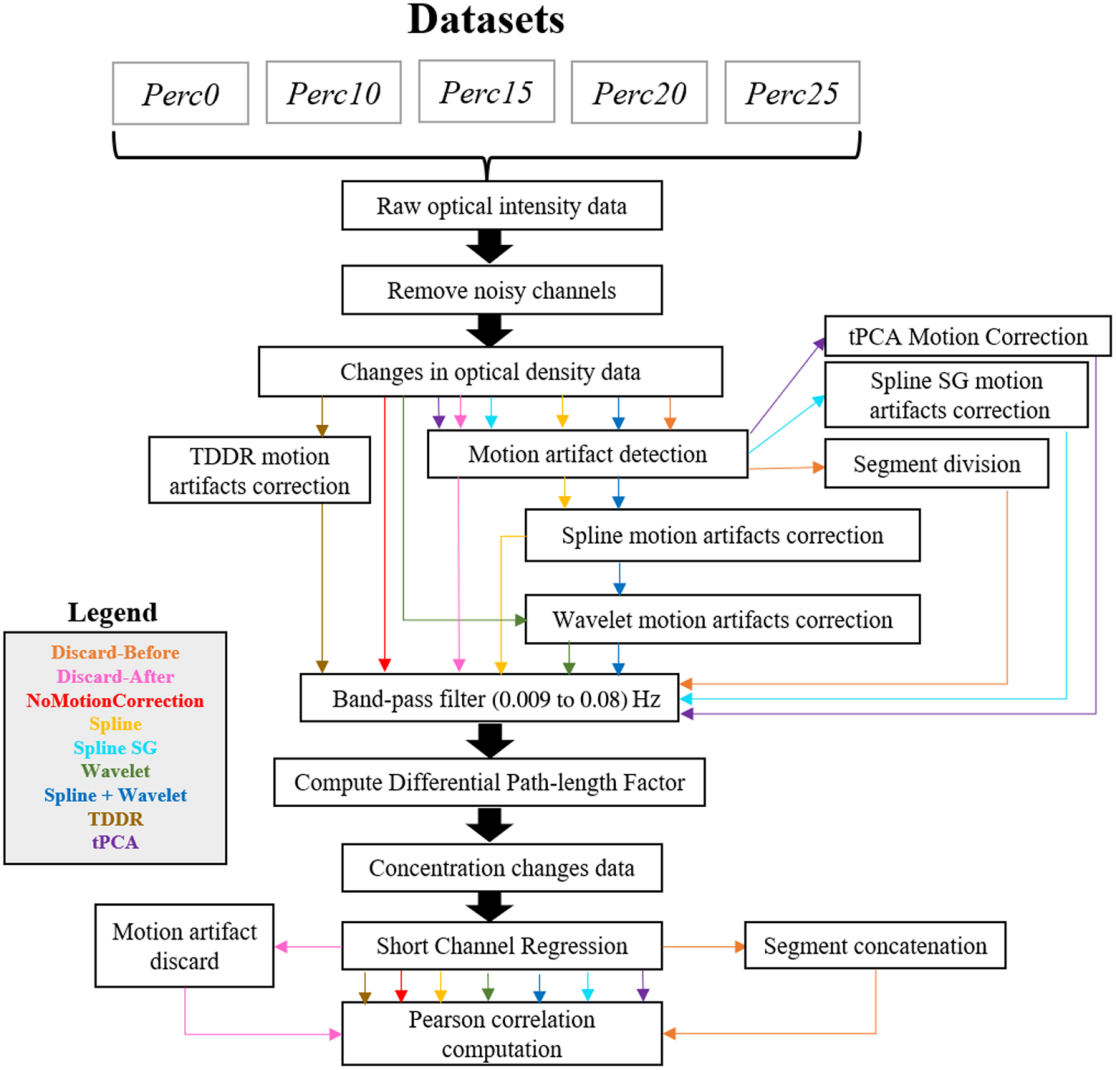
Signal processing steps for all pipelines. Each pipeline is represented by colored arrows: orange for discard-before, pink for discard-after, red for no motion correction, yellow for spline, light blue for spline SG, green for wavelet, blue for spline + wavelet, brown for TDDR, and violet for tPCA.

For each participant, the fNIRS signals were pre-processed with fifteen different pipelines (Fig. 4), which differed only in how they dealt with motion artifacts. In all pipelines, channels with a signal-to-noise ratio lower than two (SNR<2) or with a very low optical intensity were discarded. The remaining channels were converted into optical density changes. Afterward, each pipeline applied a different motion correction approach. The signal was band-pass filtered (0.009 to 0.08 Hz) and then converted into concentration changes using a subject-specific differential path-length factor.^59^ Physiological noise regression was performed to regress out the most correlated SS channel from each standard channel. At the end of each processing pipeline, the Pearson correlation between each couple of standard channels was computed for each subject, thus yielding the individual correlation matrices (50×50 matrix). The calculation of the correlation matrix was performed using only 14 out of 15 min of acquisition to use only the stable signal (the first minute was removed). The group correlation matrix was computed by averaging the individual correlation matrices.

All pipelines were applied to the Perc0 dataset. no correction, discard-before, discard-after, spline, SplineSG, wavelet, spline + wavelet, and TDDR pipelines were applied to the *Perc10R*, *Perc15R*, *Perc20R*, and *Perc25R* datasets. The discard-before, discard-after, and tPCA pipelines were applied to the *Perc10F*, *Perc15F*, *Perc20F*, and *Perc25F* datasets. tPCA was applied only to the fixed dataset because its main assumption is that MAs should be coherent across channels, and this hypothesis was not verified on the random dataset. The discard approaches were applied also to the fixed dataset because we expected its performance to be unreliable on the random dataset due to the removal of too many segments of data.

The discard-before pipeline applied to the *Perc0* dataset was considered to be the ground truth (GT), whereas all other combinations of pipelines and datasets previously described were considered to be testing pipelines.

### 2.5 Metrics of Comparison

To compare the different pipelines, three metrics were assessed: the absolute error, the slope coefficient, and the similarity of group correlation matrices. All of the metrics were computed on the group correlation matrices. Each group correlation matrix was symmetrical, and each matrix cell contained the Pearson correlation value (r-value) of the two channels associated with that cell.

The individual absolute error for each couple of channels was computed as $\mathrm{abs}(C_{x,y}^{GT}-C_{x,y})$, where $C_{x,y}^{GT}$ is the Pearson correlation coefficient between channels x and y in the ground truth dataset and $C_{x,y}$ is the Pearson correlation coefficient between the same channels for the tested dataset and pipeline. The overall absolute error was obtained by taking the median of the absolute errors of all couples of channels. Absolute errors were submitted to a repeated-measures ANOVA with the method (15 levels: discard-before, discard-after, no correction, spline, SplineSG, Wavelet15, Wavelet12, Wavelet08, Wavelet05, SplineWavelet15, SplineWavelet12, SplineWavelet08, SplineWavelet05, TDDR, and tPCA) as within the channel pair factor.

The slope coefficient was computed for each comparison of GT versus tested pipeline/dataset by performing a linear regression between the r-values of the GT and the r-values of the tested pipeline. A slope coefficient closer to one means a better match between matrices. To test the linearity between r-values, a Pearson correlation was performed. The higher the coefficient is, the more linear the relation is.

The similarity of group correlation matrices was computed by performing a non-parametric test (Wilcoxon rank-sum test, p<0.05) to evaluate statistical differences between the correlation matrices of the ground truth and the tested pipeline/dataset.

## 3 Results

### 3.1 Performance of Motion Artifact Identification Techniques

**Fig. 5:**
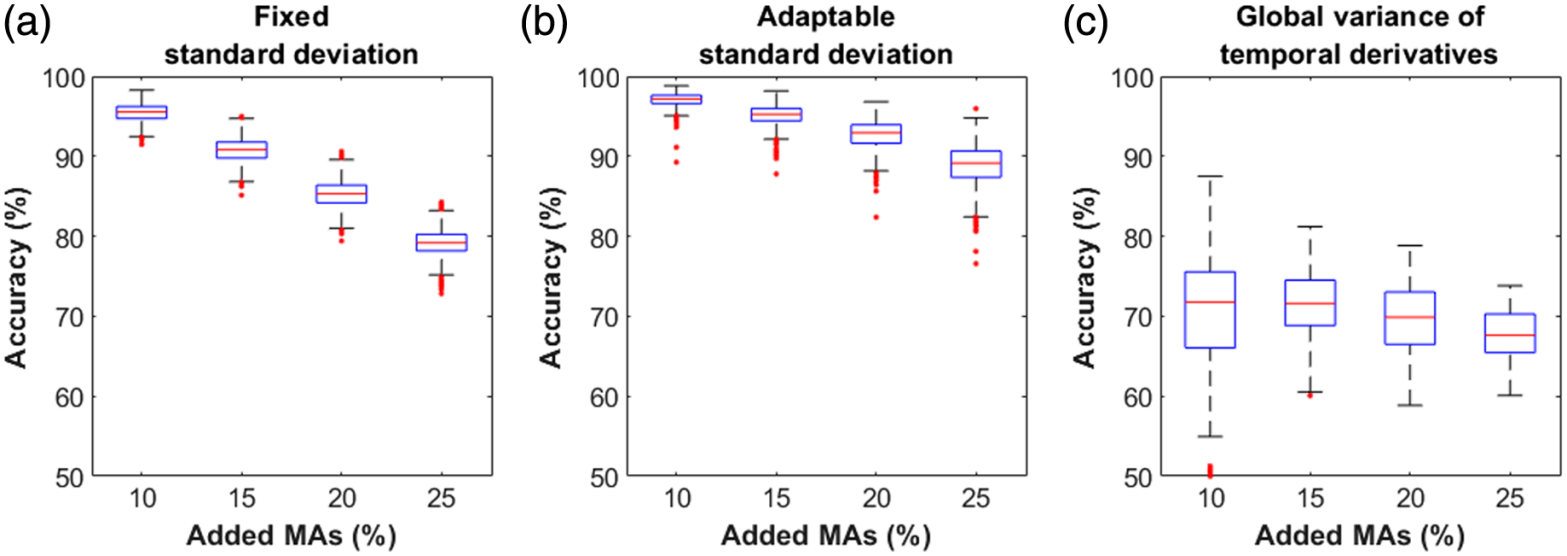
Accuracy percentage values for each detection approach increasing the level of motion artifact contamination. (a) Fixed standard deviation approach. (b) Adaptable standard deviation approach. (c) GVTD.

The average percentage of detected versus added MAs for the approach with adaptable standard deviation revealed the best performance, identifying a percentage of MAs comparable to the percentage of added MAs (10.8%, 15.8%, 20.7%, and 24.9% for *Perc10*, *Perc15*, *Perc20*, and *Perc25*, respectively), whereas the other two approaches failed in detecting the correct amount of MAs. In the fixed standard deviation approach, despite an increase in MA percentage in the different datasets, the percentage of detected MAs remained almost unchanged (7.8%, 8.3%, 8.2%, and 7.7% for *Perc10*, *Perc15*, *Perc20*, and *Perc25*, respectively), whereas for the GVTD approach, the estimation of MAs was consistently underestimated (4.2%, 4.9%, 5.6%, and 6.3% for *Perc10*, *Perc15*, *Perc20*, and *Perc25*, respectively). Figure 5 shows the accuracy of the three different motion detection approaches. The GVTD technique had the worst performance on all datasets (max value: 71.8%). For the other two approaches, increasing the percentage of added motion artifacts decreased the accuracy, with a steeper decrease for the detection technique with fixed standard deviation compared with the adaptable standard deviation one. Overall, accuracy values were higher for the adaptable standard deviation approach for all percentages of added MAs.

### 3.2 Performance of Motion Correction Techniques

#### 3.2.1 *Perc0* dataset

**Fig. 6:**
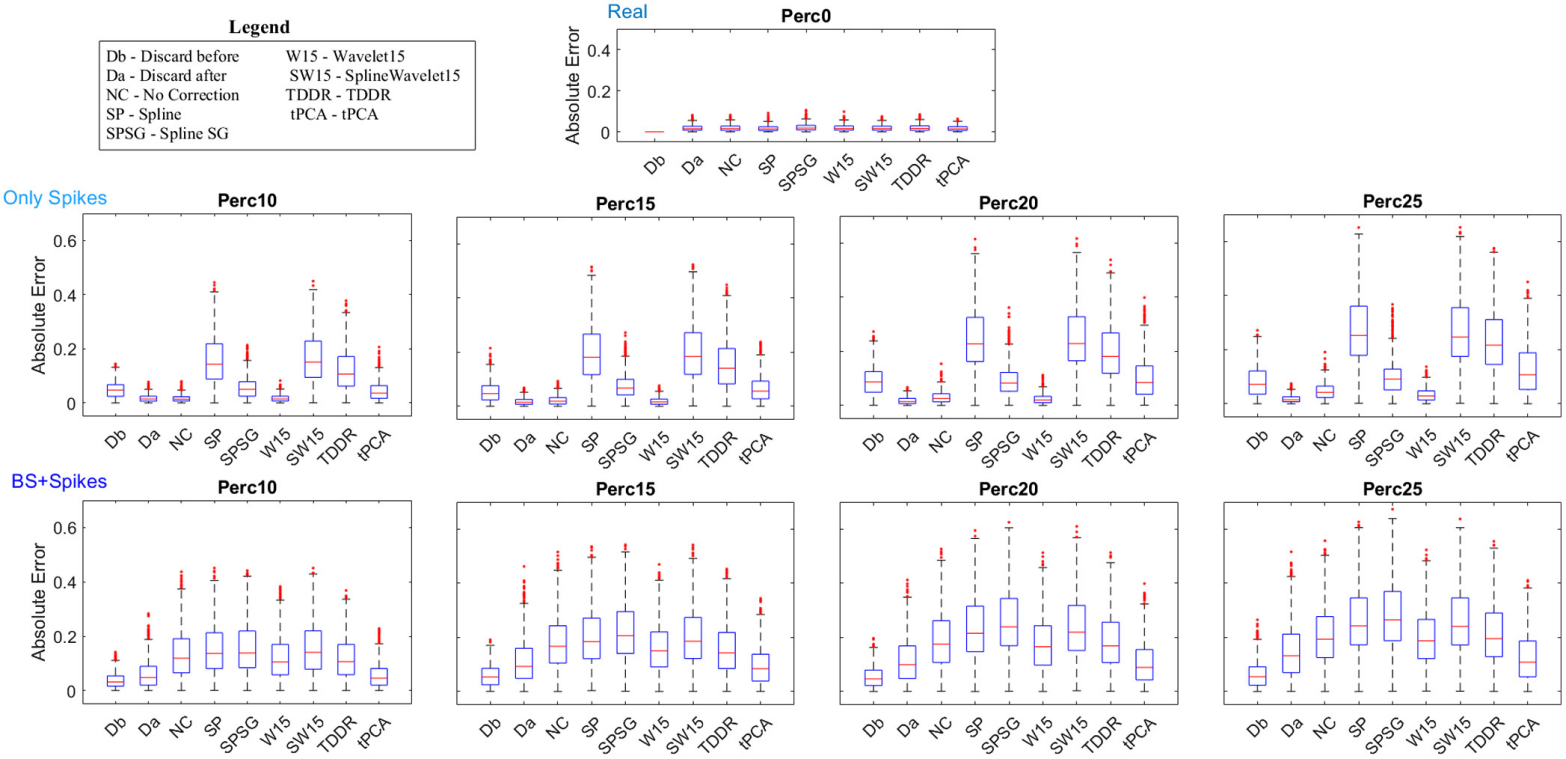
Boxplots of the individual absolute error for each dataset and pipeline. X-labels are specified in the legend box. For the techniques tested with different *iqr* values (wavelet and the combination of spline and wavelet), only the one with the best-performing *iqr* is reported.

For the *Perc0* dataset, the smallest median absolute error was obtained with the spline pipeline (0.0144), followed by tPCA (0.0147), discard-after (0.0157), wavelet with iqr=1.5, 1.2, and 0.8 (0.0167, 0.0161, and 0.0166, respectively) (Fig. 6). For the combination of spline and wavelet (SW), decreasing the *iqr* parameter increased the absolute error. The worst performance was scored by the combination of SW with iqr=0.5 (0.023). Statistically, a significant main effect was observed for the method factor. Post hoc analysis revealed that the combination of SW with iqr=0.5 differed from all other pipelines, revealing the highest absolute error. There was no statistical difference among the combination of SW with iqr=0.8, SplineSG, and wavelet with iqr=0.5. Finally, discard-after, no correction, spline, wavelet with iqr=1.5, 1.2, and 0.8, the combination of SW with iqr=1.5 and 1.2, TDDR and tPCA were found not to be statistically different.

All motion correction approaches obtained a high slope coefficient (min slope coefficient = 0.99), demonstrating good agreement between the Pearson correlation coefficients of the ground truth and the ones obtained after correction. Furthermore, the linearity was achieved by all techniques (min value = 0.98). The performance of the different pipelines for *Perc0* is shown in Fig. S3 in the Supplementary Material.

Statistically comparing the similarity of the group correlation matrices resulted in two out of thirteen testing pipelines with a correlation matrix statistically different from the ground truth: the combination of SW with iqr=0.5 (p=0.003) and TDDR (p=0.0249).

#### 3.2.2 Only spike datasets

When applied to the random dataset, the discard pipelines removed too many temporal frames, as expected, and did not allow for further analysis. Therefore, the discard pipelines were evaluated only on the fixed dataset.

For all semi-simulated datasets, the smallest median absolute error was obtained with the discard-after (*Perc10F*, 0.0146, *Perc15F*, 0.0148, *Perc20F*, 0.0144, and *Perc25F*, 0.0145) and wavelet with iqr=1.5 (*Perc10R*, 0.0145, *Perc15R*, 0.0158, *Perc20R*, 0.0202, and *Perc25R*, 0.0286) pipelines. The wavelet pipeline increased the absolute error as the degree of MA contamination increased and with the decrease of the *iqr* parameter, but the highest absolute error remained less than 0.039 (see Fig. 6). The no correction performance was in line with the wavelet performance, whereas discard-before (*Perc10F*, 0.047, *Perc15F*, 0.047, *Perc20F*, 0.087, *Perc25F*, 0.072), SplineSG (*Perc10F*, 0.050, *Perc15F*, 0.067, *Perc20F*, 0.083, *Perc25F*, 0.091), and tPCA (*Perc10F*, 0.036, *Perc15F*, 0.056, *Perc20F*, 0.084, *Perc25F*, 0.107) increased the error. For the TDDR, the spline and the combination of SW the median absolute error increased dramatically. The worst performance was scored by the combination of SW with iqr=0.5 (*PercR10*, 0.178; *PercR15*, 0.206; *PercR20*, 0.248; *PercR25*, 0.261). For all datasets, ANOVA showed a significant main effect. A similar pattern of results was observed across all datasets, allowing us to identify four distinct groups of data processing pipelines: discard-after, no correction, and wavelet differed from all other pipelines; tPCA, SplineSG, and discard-before differed from all other pipelines; TDDR differed from all other pipelines; and spline and the combination of SW differed from all other pipelines. Within groups, in the *Perc10* and *Perc15* datasets, post hoc analysis showed no statistically significant differences among the discard-after, no correction, and wavelet pipelines. However, in the *Perc20* dataset, discard-after statistically differed from wavelet with an *iqr* of 0.5, and in the *Perc25* dataset, discard-after statistically differed from no correction and wavelet with *iqr* values of 1.2, 0.8, and 0.5. Additionally, tPCA and discard-before showed differences from all other methods in the *Perc15*, *Perc20*, and *Perc25* datasets and from SplineSG in all datasets. Finally, in the *Perc10* dataset, spline statistically differed from the combination of SW with *iqr* values of 0.8 and 0.5. In the *Perc15* and *Perc20* datasets, spline statistically differed only from the combination of SW with an *iqr* of 0.5. However, in the *Perc25* dataset, spline did not exhibit any significant differences from the combination of SW.

For the *Perc10* dataset, the discard-after, no correction, and wavelet pipelines obtained a high slope coefficient (min slope coefficient = 1.04, min linearity coefficient = 0.98) (Fig. S4 in the Supplementary Material). The best-performing pipeline was the wavelet with iqr=1.5 pipeline, which achieved a perfect matching with the ground truth (slope coefficient = 1). Discard-before, SplineSG, and tPCA obtained a good slope coefficient (0.87, 0.82, and 0.73, respectively), TDDR was less performant (0.46), and spline and the combination of SW obtained the worst slope coefficients (min slope coefficient = 0.33; max slope coefficient = 0.37) (see Fig. S12 in the Supplementary Material).

For *Perc15*, *Perc20*, and *Perc25*, the main pattern remained the same as the *Perc10* dataset: the best performance was achieved by discard-after pipeline followed by wavelet and no correction pipelines; a good performance was also obtained by the discard-before pipeline followed by the SplineSG pipeline, which had a fair slope coefficient. The tPCA pipeline exhibited a reasonable slope coefficient only for the Perc15 dataset, whereas the worst performance was obtained by the combination of SW pipeline with iqr=0.5 followed by the combination of SW with higher *iqr*, spline, and TDDR pipelines (see Fig. 6, see Figs. S5–S7 and S13–S15 in the Supplementary Material).

The linearity coefficient remained high for all technique datasets except for *Perc25*, which lost linearity for spline and the combination of SW pipeline (min linearity coefficient value: Perc10, 0.84; *Perc15*, 0.82; *Perc20*, 0.81; *Perc25*, 0.39). Overall, although the slope coefficient decreased, the main pattern remained similar. However, when the slope coefficient decreased under 0.3, the main pattern was lost. It should be noted that we reported only positive r-values because all negative values were approximately equal to zero. Looking at the group correlation matrices obtained from the fifteen pipelines applied to the nine different datasets, less than 0.001% of the r-values had negative values, and none of them were lower than –0.05.

In the *Perc10* dataset, nine out of fifteen testing pipelines showed a statistical difference from the ground truth: discard-before (p<0.001), spline (p<0.001), SplineSG (p<0.001), wavelet with iqr=0.5 (p=0.013), the combination of SW with iqr=1.5, 1.2, 0.8 and 0.5 (p<0.001), and TDDR (p<0.001). The correlation matrix obtained after applying the other pipelines did not statistically differ from the ground truth correlation matrix. In the *Perc15* dataset, twelve out of fifteen testing pipelines showed a statistical difference from the ground truth: discard-before (p<0.001); spline (p<0.001); SplineSG (p<0.001); wavelet with iqr=0.8 and 0.5 (p=0.007 and p<0.001); the combination of SW with iqr=1.5, 1.2, 0.8, and 0.5 (p<0.001); TDDR (p<0.001); and tPCA (p<0.001). The correlation matrix obtained after applying the other pipelines did not statistically differ from the ground truth correlation matrix. Finally, in the *Perc20* and *Perc25* datasets, all pipelines except for discard-after differed from the ground truth (p<0.01).

#### 3.2.3 BS+Spikes datasets

For all semi-simulated datasets, the smallest median absolute error was obtained with the discard-before (*Perc10F*, 0.032, *Perc15F*, 0.053, *Perc20F*, 0.046, and *Perc25F*, 0.054) pipeline followed by tPCA (*Perc10F*, 0.046, *Perc15F*, 0.084, *Perc20F*, 0.089, and *Perc25F*, 0.108) and discard-after (*Perc10F*, 0.048, *Perc15F*, 0.093, *Perc20F*, 0.099 and *Perc25F*, 0.132). The wavelet pipeline increased the absolute error as the degree of MA contamination increased and with the decrease of the *iqr* parameter (min value: 0.105; max value: 0.203). No correction and TDDR performance was in line with wavelet with iqr=0.8 and 0.5, whereas spline and the combination of SW increased the median absolute error. The worst performance was scored by the combination of SW with iqr=0.5 (*PercR10*, 0.165; *PercR15*, 0.208; *PercR20*, 0.239; *PercR25*, 0.258) (see Fig. 6). For all datasets, ANOVA showed a significant main effect of the method factor. Post hoc analysis revealed that discard-after, discard-before, and tPCA statistically differed from all other pipelines in each dataset. Furthermore, discard-before significantly differed from tPCA in all datasets and from discard-after in the *Perc15*, *Perc20*, and *Perc25* datasets, whereas discard-after and tPCA did not differ in any dataset. All statistical results are reported in Supplementary Material (see Table S1 in the Supplementary Material).

For all datasets, the highest slope coefficient was obtained by the discard-before pipeline (Perc10, 0.86; Perc15, 0.77; Perc20, 0.81; Perc25, 0.75). Discard-after and tPCA obtained a fair slope coefficient. Spline, SplineSG, TDDR, Wavelet, and the combination of SW decreased the slope coefficient, and the combination of SW with iqr=0.5 obtained the worst slope coefficients (*PercR10:* 0.30; *PercR15*, 0.22; *PercR20*, 0.14; *PercR25*, 0.10) (see Figs. S8–S15 in the Supplementary Material).

In all datasets, all testing pipelines showed a statistical difference from the ground truth (p<0.001).

**Fig. 7:**
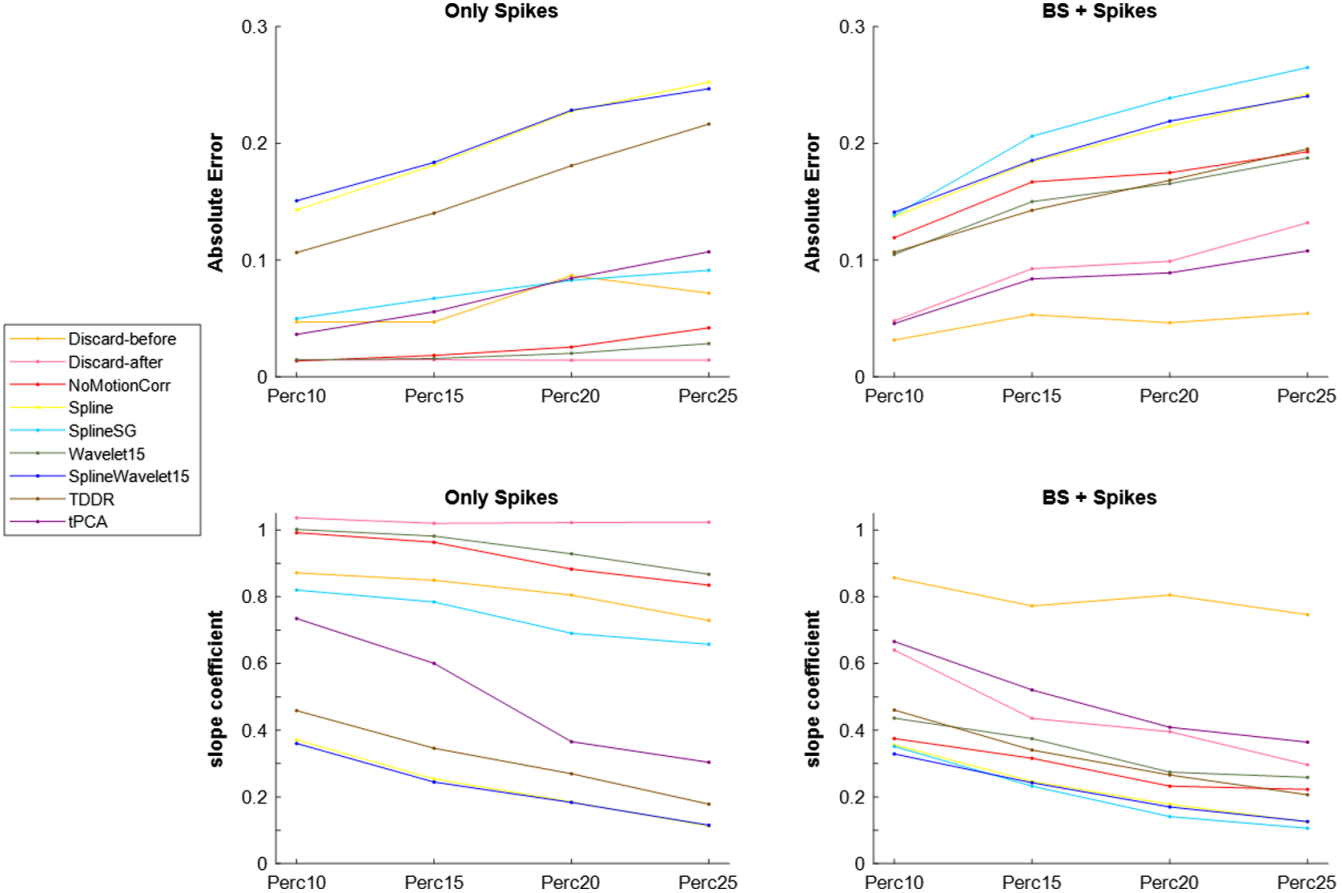
Summary of the absolute errors (at the top) and the slope coefficients (at the bottom) as a function of the percentage of motion artifacts is presented for both the Only Spikes dataset (on the left) and the BS+Spikes dataset (on the right).

Figure 7 provides a summary of both the absolute error and the slope coefficient for the Only Spikes and BS+Spikes datasets.

Optical Density signals before and after different motion correction techniques are shown in Supplementary Material (see Fig. S16 in the Supplementary Material).

## 4 Discussion

Several papers have been published in the past years comparing motion correction techniques on task-based data, but little is known about their performance on resting-state data. In this work, we pursued what could be the best approach to clean resting-state data from motion artifacts to provide guidelines for users depending on the degree and typology of motion artifact contamination of their data. We found that the optimal strategy for addressing motion artifacts (MAs) in resting-state data is contingent upon the specific nature of the artifacts present in the dataset. As a general rule, the most effective solution entailed the removal of frames affected by motion artifacts. Additionally, when the dataset predominantly contained spike-type artifacts, if discarding segments of artifacts would not be a suitable method for the users’ aim (e.g., too short acquisition or dynamic functional connectivity analysis), the preferred correction method resulted in wavelet filtering with a suitable *iqr*. Instead, in cases in which both spike-type and baseline shift-type MAs were prevalent, the most suitable alternative to the discard approach was tPCA. These findings are not in line with the guidelines suggested for task-based data, thus enhancing the importance of treating these two data types differently.

To provide guidelines as a function of the degree of motion artifact contamination, in addition to the real dataset, four other different datasets were created with a semi-simulated process: the random and the fixed datasets for the Only Spikes and BS+Spikes datasets. The spline and wavelet techniques work on a single-channel basis (meaning that artifacts could be temporally independent in each channel), whereas tPCA relies on the simultaneity of MAs in most of the channels. The fixed dataset was created to provide tPCA with a sound framework to evaluate its performance and to properly evaluate the discard pipelines, for in the random dataset, too many frames had to be removed as the degree of contamination increased (being the artifacts temporally independent). Overall, an advantage of all datasets was that most of the introduced MAs were real and not modeled. We created both the Only Spikes and the BS+Spikes datasets to provide a better understanding of how to deal with different types of motion artifacts.

In the semi-simulated datasets, the amount and location of artifacts are known. Thus, we were able to test the performance of three MA identification techniques. Optimal MA identification is essential because some motion correction techniques are applied only to the frames identified as motion artifacts. If the motion artifact is not correctly identified, then the performance of that motion correction technique could be biased. To put forth a fair comparison between different methods, a reliable identification technique was required. Yang et al. introduced a novel motion identification technique that is suitable for datasets highly contaminated by motion artifacts. The percentage of MAs in their dataset was around 25% to 35%. Yang and colleagues demonstrated a higher detection efficiency with an adaptable standard deviation compared to the standard Homer approach, which has a fixed standard deviation. Sherafati et al.^55^ proposed a new motion detection method for multi-channel optical imaging systems that leverages spatial patterns across measurement channels. Here, we compared the performance of the three motion detection techniques on datasets with different percentages of MAs (10%, 15%, 20%, and 25%). Results showed that the percentage of added MAs and MAs detected in each channel by the fixed standard deviation technique was not comparable in three out of four percentage conditions. Regardless of the added percentage, the fixed standard deviation approach always detected a percentage of around 10%. The adaptable standard deviation approach, instead, always approached the correct inserted percentage regardless of the contamination degree. The GVTD approach failed in detecting MAs in all datasets, always identifying an MA percentage of less than 10%. It is worth emphasizing that the performance of the GVTD approach is likely underestimated, given that the dataset, with artifacts inserted in random positions within each channel, is not ideal for the GVTD approach.

Moreover, the accuracy value suggested a better performance for the adaptable than the fixed standard deviation and GVTD approaches. Yang and colleagues tested their approach on infant datasets that are heavily contaminated by MAs. In our work, we ranged from 10% to 25% of contamination, under the hypothesis that the number of motion artifacts is expected to be smaller if data come from an adult population. Our comparison demonstrated that the adaptable standard deviation approach is suitable and ideal with datasets with different contamination of MAs. The innovative approach avoids the computation of a biased standard deviation, which is essential for motion artifact identification. When the number of artifacts in the signal increases, the standard deviation computed over the entire signal (fixed standard deviation approach) moves further away from the true value. Instead, extracting the least noisy frames from each signal time window leads to calculating the standard deviation only on the part of the signal free of motion artifacts, approaching the true standard deviation of the signal. Because motion correction functions, which require a previous motion identification step, are highly dependent on the motion identification performance, an increasingly precise technique is required. Considering our results, which confirm Yang’s results, the actual standard deviation threshold implemented in Homer2/3 should be updated to make it adaptable. 

The main aim of this paper was to find the best solutions to pre-process resting-state fNIRS data as a function of the degree and typology of MA contamination. To achieve this aim, three different metrics were evaluated: the absolute error (the lower the value is, the higher the match between r-values of GT and the tested pipeline is), the slope coefficient (the higher the value is, the better the performance is), and the similarity coefficient, a statistical parameter.

The discard-before approach applied to the *Perc0* dataset was used as the ground truth. In the Only Spikes datasets, the discard-after approach resulted in the best solution with the lowest absolute error, the highest slope coefficient, and no statistical difference from the GT. For the BS+Spikes datasets, instead, the discard-before approach proved to be the most effective, showcasing the lowest absolute error and the highest slope coefficient. It is worth noting that, in this work, the highest percentage of MAs in the dataset was 25% and the acquisition lasted 15 min. Thus, removing 25% of the signal still leaves a reasonable number of frames (about 10 min), which has been shown to be sufficient for a reliable correlation matrix computation. In a study by Wang et al.,^60^ they employed graph theory metrics to determine that achieving accurate and stable functional connectivity (FC) required a minimum fNIRS imaging duration of 7.0 min at high network thresholds, whereas at low network thresholds, the necessary scanning time was reduced to a minimum of 2.5 min. Because the minimum duration for valid resting-state parameters is around 5 min, the discard method could be valid when the removal of artifactual frames still leaves a trace of at least 5 min.^61^ These results are in line with the results by Selb and colleagues,^30^ which suggested the discard approach as the best approach to deal with motion artifacts in IHC analysis. Although the discard method is suitable for studying functional connectivity (if enough data are kept after the discard approach), it is not ideal when the aim is to study dynamic functional connectivity. The discard approach is not a valid method because removing frames, independently in each channel, creates temporal holes, generating discontinuities in the signal.

In this situation, in the Spike Only dataset, applying motion correction with wavelet with high *iqr* (1.2 to 1.5) could be a good alternative. Wavelet maintains all temporal frames, and the error achieved after applying wavelet in our dataset was the lowest among the motion correction pipelines. The wavelet method is applied to the entire time series; therefore, the choice of the *iqr* parameter has a significant impact on the results. If the *iqr* is too low, the function may delete wavelet detail coefficients associated with lower frequency content, which might be essential components for the computation of the correlation matrix. These results highlight the importance of reporting all parameters employed in the analysis because the same procedure with different parameters can yield very different results.

Surprisingly, the no correction pipeline yielded a good performance. The band-pass filter was applied in the [0.008 to 0.09] Hz range, which is lower than the typical range used in task-based studies ([0.01 to 1.5] Hz). Most motion artifacts have a high-frequency content, and therefore, their contribution to the data can likely be completely removed by the filter itself. This is not occurring in task-based studies because the higher frequency range of the filter is not enough for their removal. Another reason that the no correction pipeline performance was good could be due to the presence of short separation channels in our study. Regressing short separation channels, where motion artifacts were present as well, could have actually not only reduced physiological oscillations but also inherently corrected the motion artifacts. However, we deem this second hypothesis less likely because the no correction pipeline was tested on the random dataset, for which motion artifacts were not temporally correlated among channels. In most fNIRS systems, the number of available SS channels is limited; therefore, it is highly likely that motion artifacts present in standard channels might not be present in SS channels, above all when these are far away from each other, and vice versa. In novel high-density systems that encompass several SS channels widely spread over the head, it is highly likely instead that SS channels and standard channels share the same motion artifacts, thus making it possible to use the no correction approach with SS regression as a likely valid solution for motion correction.

In the BS+Spikes dataset, when the discard-before approach is not suitable, the best alternative is the tPCA approach. The discard-after approach is as good as the tPCA one, but it does not allow for the study of dynamic functional connectivity. It is noteworthy that the tPCA method exhibits a nearly identical behavior across the two distinct dataset types (with and without baseline shift artifacts). However, in the case of the Only Spikes datasets, other motion correction approaches performed better than tPCA, which is therefore not considered the ideal approach. Generally speaking, baseline shift artifacts seem therefore to be more challenging to correct, with results that diverge further from the ground truth independently of the applied technique. Their presence, therefore, poses increased challenges for achieving robust signal decontamination.

In the BS+Spikes dataset, tPCA resulted in the best motion correction approach, whereas in the Only Spikes dataset, tPCA yielded a worse performance than Wavelet, but a better one than spline and the combination between spline and wavelet. We acknowledge that, although the fixed dataset adheres to the assumptions of tPCA by ensuring temporally correlated motion artifacts composing the majority of signal variation, it represents an idealistic scenario. In real-world data, motion artifacts may not be present in all channels and could exhibit slight temporal delays across different channels. This deviation from real data characteristics may impact the performance of multivariate methods that rely on spatial filtering. Therefore, tPCA performance might have been overestimated in this work. It is also worth noting that, even in an ideal situation, for both Only Spikes and Spikes+BS datasets, the tPCA approach yielded group correlation matrixes statistically different from the GT. It is likely that the variance removed with this technique was not only due to motion artifacts but also to slow oscillations, which might have been important for RSFC computation. In the BS+Spikes dataset, wavelet and no correction are not achieving the same good performance obtained in the Only Spikes dataset. For the no correction approach, baseline shifts might be the most challenging artifacts because, although their high-frequency contribution might be removed by the filter, they might introduce some low-frequency content due to the baseline shift not being corrected. Furthermore, wavelet is known to be unable to correct baseline shifts.

In both Spike Only and BS+Spikes datasets, our results show that spline and the combination of spline and wavelet are not a good choice to pre-process resting-state data. It is likely that the spline interpolation introduced low-frequency components in the signal, which created a bias when the functional correlation matrix was computed. The impact of these spurious components is higher as the number of artifacts increases, with spline worsening its performance as the degree of motion artifact contamination increases. The negative performance of the combination of spline and wavelet is a direct consequence of the biased performance of the spline approach.

The Spline-SG approach achieved the worst performance among methods in the BS+Spikes dataset, whereas it had a performance similar to tPCA and discard-before in the Only Spikes dataset. Increasing the motion artifact contamination in the datasets decreases the SNR of the signal. In the Spline-SG approach, the spline interpolation step is applied only when the SNR < 3, which is almost never the case with data highly contaminated by MAs. Therefore, most of the time, only the SG part of the approach was applied. This is probably why Spline-SG showed the worst performance in the BS+Spikes dataset as smoothing out the baseline shifts caused the introduction of low-frequency biases in the signal. The average performance in the Spikes Only dataset is due to the good performance of the smoothing approach on high-frequency spikes, introducing less biases than in the dataset with baseline shifts as well.

TDDR performance is similar across datasets. In the Only Spike dataset, TDDR performance is one of the worst, performing a bit better than spline and the combination of spline and wavelet but worse than all other pipelines. In the BS+Spikes dataset, TDDR performance is similar to the wavelet approaches. These results highlight that the TDDR performance is stable and reliable independently of the typology of MAs but that the TDDR correction might introduce some low-frequency biases that could be problematic when computing RSFC.

Although the error metric demonstrated a detrimental effect of motion correction techniques on the correlation values between channel pairs, the slope coefficient and the similarity index showed that, for most techniques, this is only reflected in a linear and homogeneous decrease in the computed r-values, maintaining the original spatial pattern of correlations. This highlights that, in most of the cases, qualitatively looking at the group correlation matrixes, researchers would have drawn the same conclusions. From a statistical perspective, though, reducing the correlation values and increasing the variance (i.e., the variability among channel pairs and subjects) could lead to different quantitative results when comparing, for example, correlation matrixes from different populations or acquisitions. The higher the variance is and the smaller the values are, the lower the probability of obtaining statistically significant results is. For the worst-performing motion correction techniques at a high degree of motion artifact contamination, the main spatial pattern of correlation was completely lost. This result was supported by the slope coefficient. The lower the coefficient is, the narrower the interval is. Thus, the lower the slope coefficient is, the higher the probability of losing the main spatial pattern is.

Selb and colleagues^30^ reported that the best approach to pre-processing motion artifact for resting state measurements is the one that discards temporal frames. These results are in line with our work. However, they proposed the spline interpolation as a good alternative, but we found this to be not recommended. A possible explanation is that they applied the identification method implemented in Homer2/3, which we have shown to be less performant than the one that we used (adaptable standard deviation identification approach). The motion identification technique used by Selb et al. might have biased the spline interpolation performance. In fact, if MAs were not correctly identified, then the spline interpolation was not adequately evaluated. Moreover, wavelet and no correction are not advised in their conclusions as in our BS+Spikes dataset. It is likely that their dataset also contained baseline-shift motion artifacts. Moreover, two other possible reasons could explain the different results from our Only Spikes dataset. First, Selb and colleagues did not use the same band-pass filter; second, they did not perform the SS channel regression. No correction and wavelet may have been diverted by physiological noise that was not correctly removed due to the lack of short-separation channel regression. Moreover, the filter could have impacted the no correction approach. Selb and colleagues used four different band-pass filters; we compared our results only with the very low-frequency oscillations one, which ranged between 0.01 and 0.07 Hz because it was closest to ours (0.008 to 0.09 Hz). However, the lower limit of the very low-frequency oscillations range was higher than the one that we used, probably leaving residual motion artifacts within the signal. This evidence underlines the relevance of choosing the band-pass filter setting when performing the no correction approach.

This study has a few limitations. Here, we investigated RSFC in a compliant healthy adult population. However, fNIRS may be a valuable tool for depicting RSFC in people with neurological diseases. This is the main reason that we semi-simulated datasets with higher percentages of MA contamination. One limitation of our study might be that the type of MAs in patients’ data might differ from the type of MAs that we extracted from the healthy adult dataset. Thus, further studies may evaluate whether MA types differ among populations. The final aim of RSFC analysis, furthermore, is usually to evaluate the presence of networks. Here, we only investigated the impact of motion correction on the estimation of the correlation matrices. It would be valuable for future research to explore the impact of correction methods on the detectability of the networks. Another limitation of this work is that the newly proposed multivariate approaches for motion correction,^62^ which have been tested on task-based data and require additional measures (e.g., accelerometers), have not been considered in the comparison. It would be interesting in the future to assess their impact on RSFC analysis compared with standard approaches.

## 5 Conclusion

Guidelines for motion correction in task-based studies suggest that correcting for motion artifacts is always the best choice. In these studies, wavelet and the combination of spline and wavelet when the degree of motion artifact contamination is substantial were found to be promising approaches. Our results suggest different guidelines for motion correction in resting-state studies, with particular attention to the type and amount of artifacts present in the dataset. Specifically, when the dataset predominantly contains spike artifacts, the optimal solution is to discard contaminated segments of data at the end of the pre-processing. As a second option, the best motion correction approach is wavelet with an iqr≥1.2 or leaving the data as they are (no correction). However, if baseline shifts are also present in the dataset, discarding contaminated segments of data at the beginning of the pre-processing is preferable. As possible alternatives, the tPCA pipeline or discarding motion artifacts at the end of the pre-processing is suitable, keeping in mind that the error introduced with the correction when baseline shifts are present is higher than when only spikes are present in the dataset. A similarity between task-based and resting-state guidelines for motion correction is that, in both cases, the impact of the technique on the recovery of the underlying data might be dependent on the degree of motion artifact contamination. When few motion artifacts are present, our results show that the impact of motion correction techniques on RSFC computation is limited, with all pipelines yielding very similar results. Thus, we recommend, as the first important step, to inspect the acquired data to be aware of the type, quantity, and temporal distribution of MAs, to select the best pipeline to be applied and whether the choice of the pipeline would be impactful or not on the results.

## Supplementary Material



## Data Availability

The data that support the findings of this study are available from the corresponding author upon request.
