# Supplementary material for: Comparing different motion correction approaches for resting-state functional connectivity analysis with functional near-infrared spectroscopy data
Source: Neurophotonics. 2024 Oct 3;11(4):045001. doi: 10.1117/1.NPh.11.4.045001 (PMC11448702; doi:10.1117/1.NPh.11.4.045001)
Supplement: Supplementary file 1 [file NPh_011_045001_SD001.docx]

**Supplementary Materials**

**
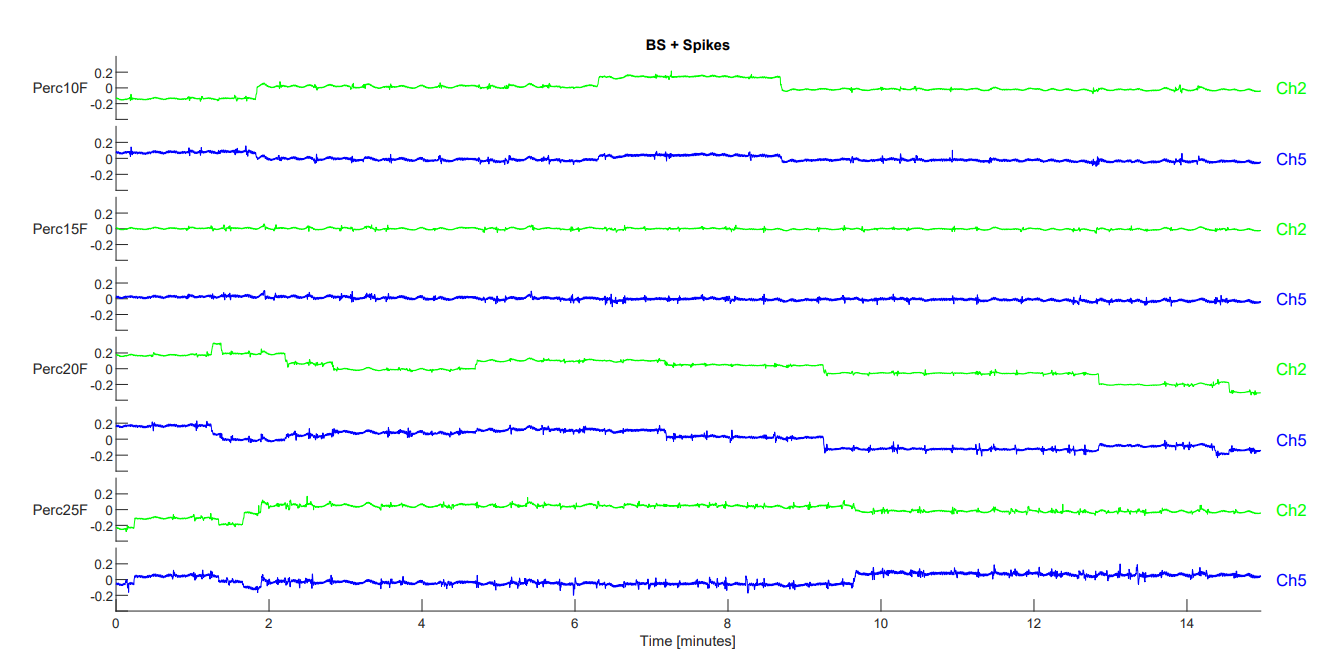
Fig. S1** Representative couple of channels for the dataset built adding MAs in fixed positions across channels in each participant (Fixed dataset). The semi-simulated datasets at 10%, 15%, 20% and 25% are displayed for the BS+Spikes dataset.

**Fig. S2** Temporal frames identified as motion artifacts (in red) by the Adaptable standard deviation algorithm in different datasets conditions. From the top to bottom: BS+Spikes dataset with 10% of added MAs, OnlySpikes dataset with 15% of added MAs, BS+Spikes dataset with 20% of added MAs and OnlySpikes dataset with 25% of added MAs.


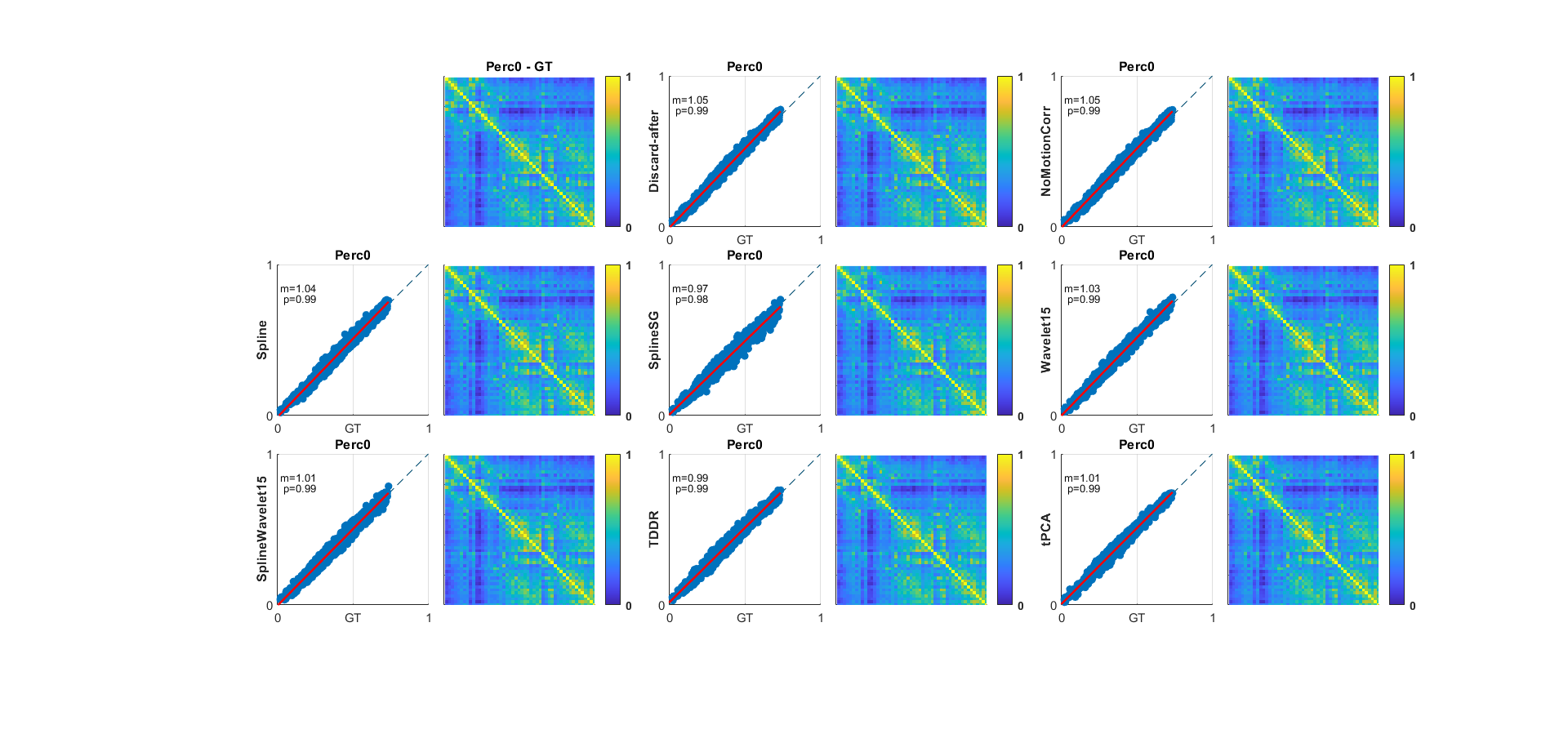


**Fig. S3** Scatterplots of testing and GT group correlation matrix in *Perc0* dataset and the correspondent group correlation matrix of the testing pipeline. All r-values range between 0 and one.


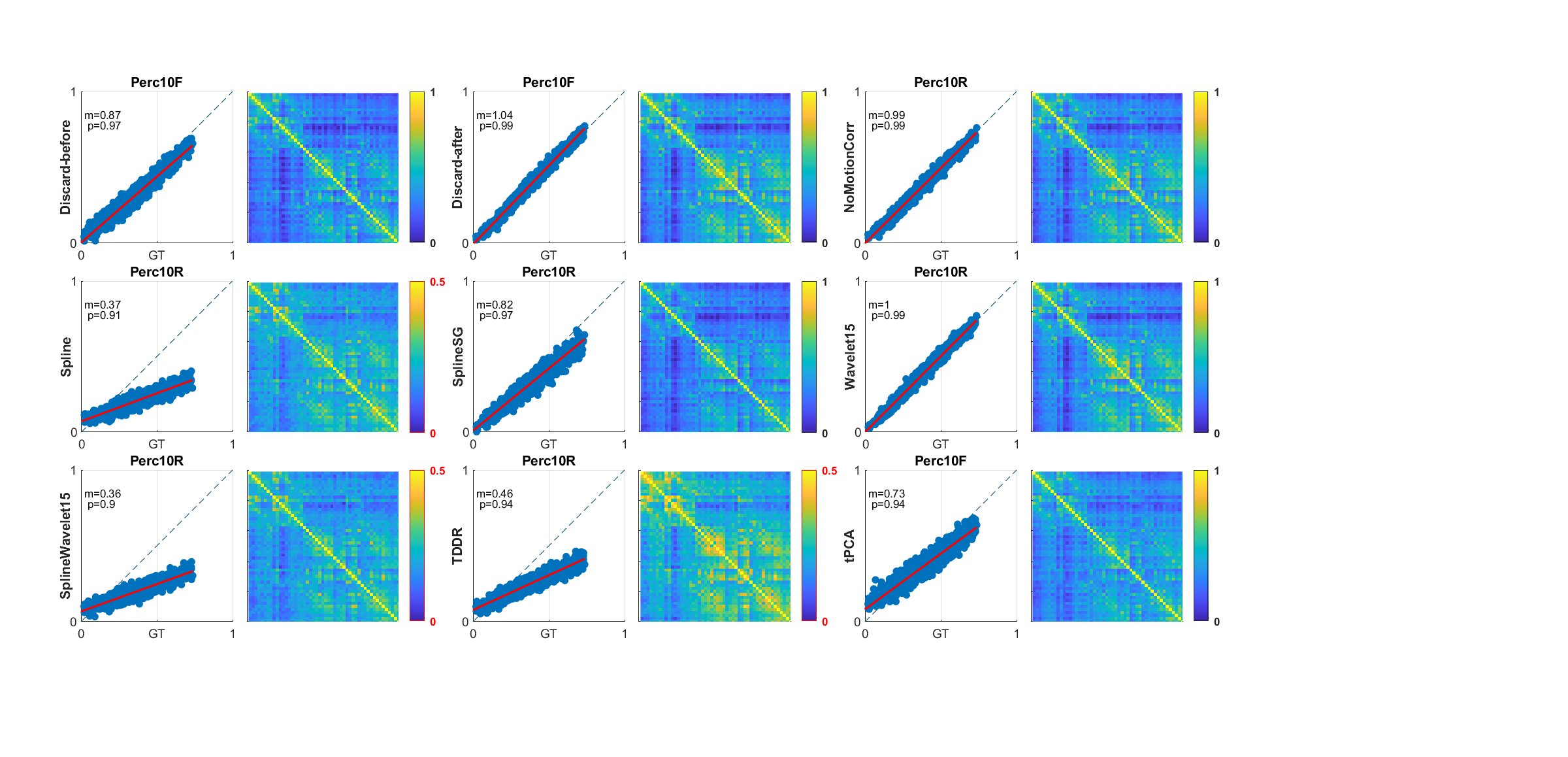


**Fig. S4** Scatterplots of testing and GT group correlation matrix in *Perc10* Only Spikes dataset and the correspondent group correlation matrix of the testing pipeline. The real color range is between zero and one. In specific cases, the range was reduced to highlight the testing correlation matrix pattern (please note the red values next to the color bars).


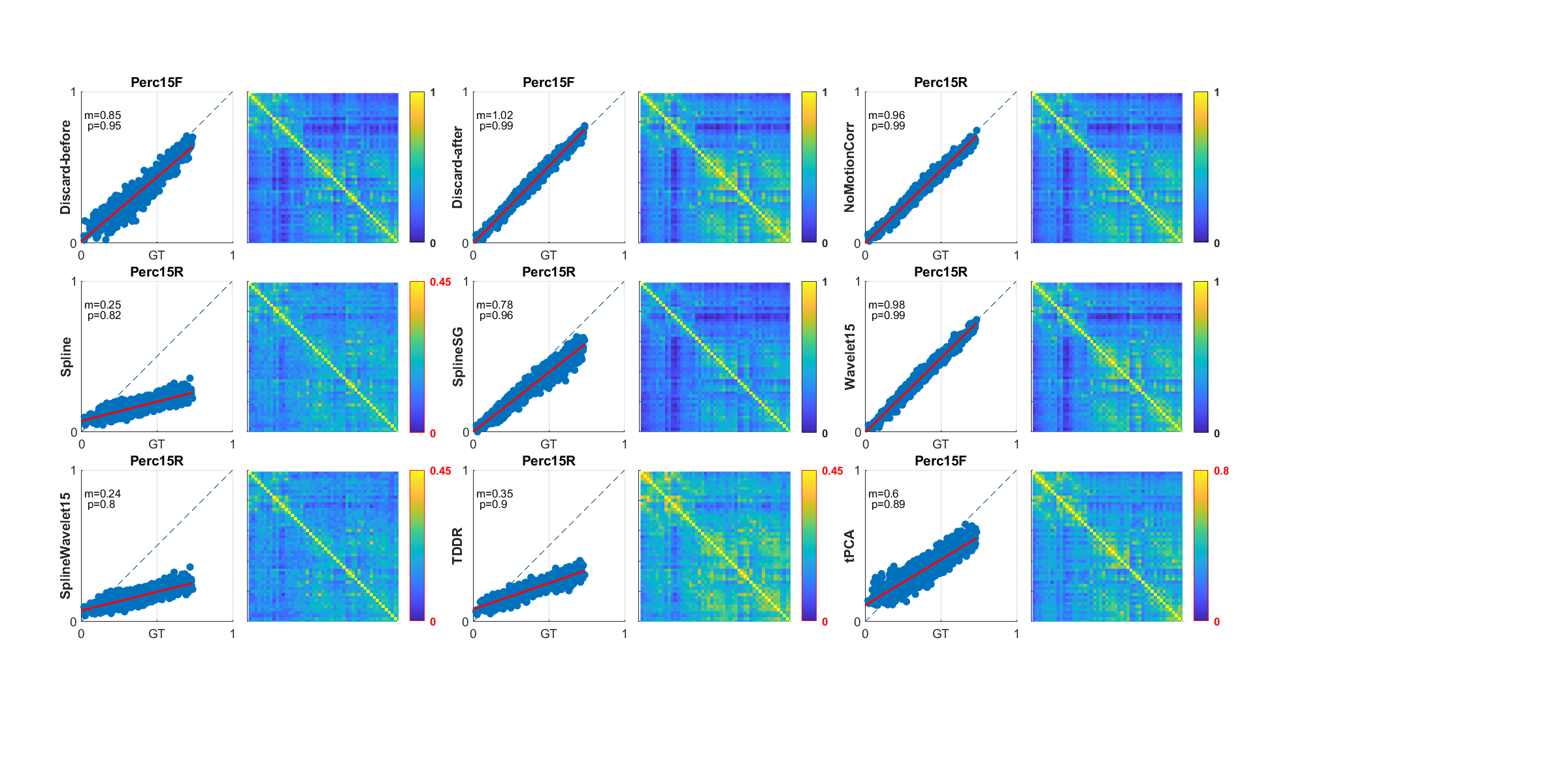


**Fig. S5** Scatterplots of testing and GT group correlation matrix in *Perc15* Only Spikes dataset and the correspondent group correlation matrix of the testing pipeline. The real color range is between zero and one. In specific cases, the range was reduced to highlight the testing correlation matrix pattern (please note the red values next to the color bars).


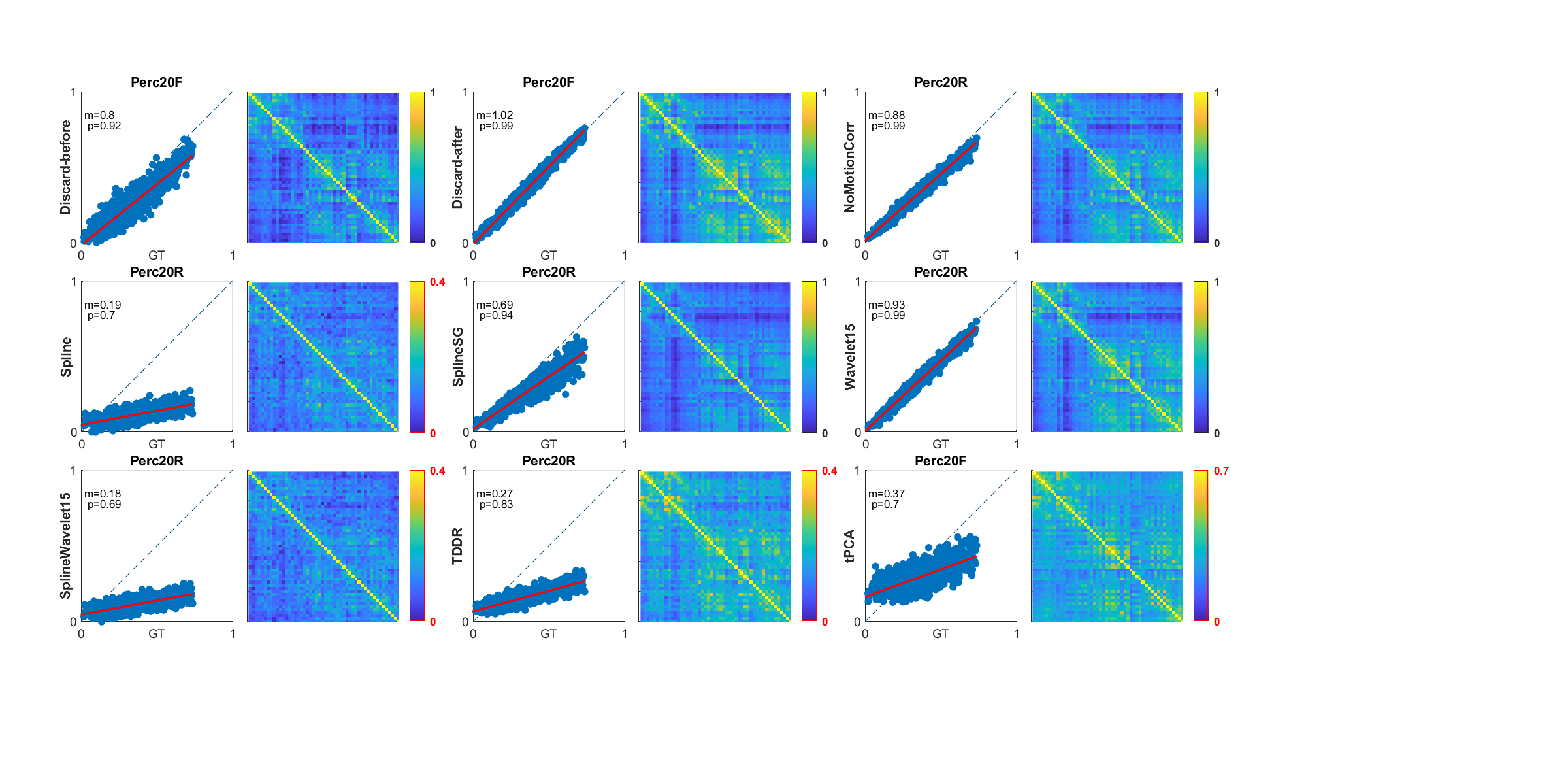


**Fig. S6** Scatterplots of testing and GT group correlation matrix in *Perc20* Only Spikes dataset and the correspondent group correlation matrix of the testing pipeline. The real color range is between zero and one. In specific cases, the range was reduced to highlight the testing correlation matrix pattern (please note the red values next to the color bars).


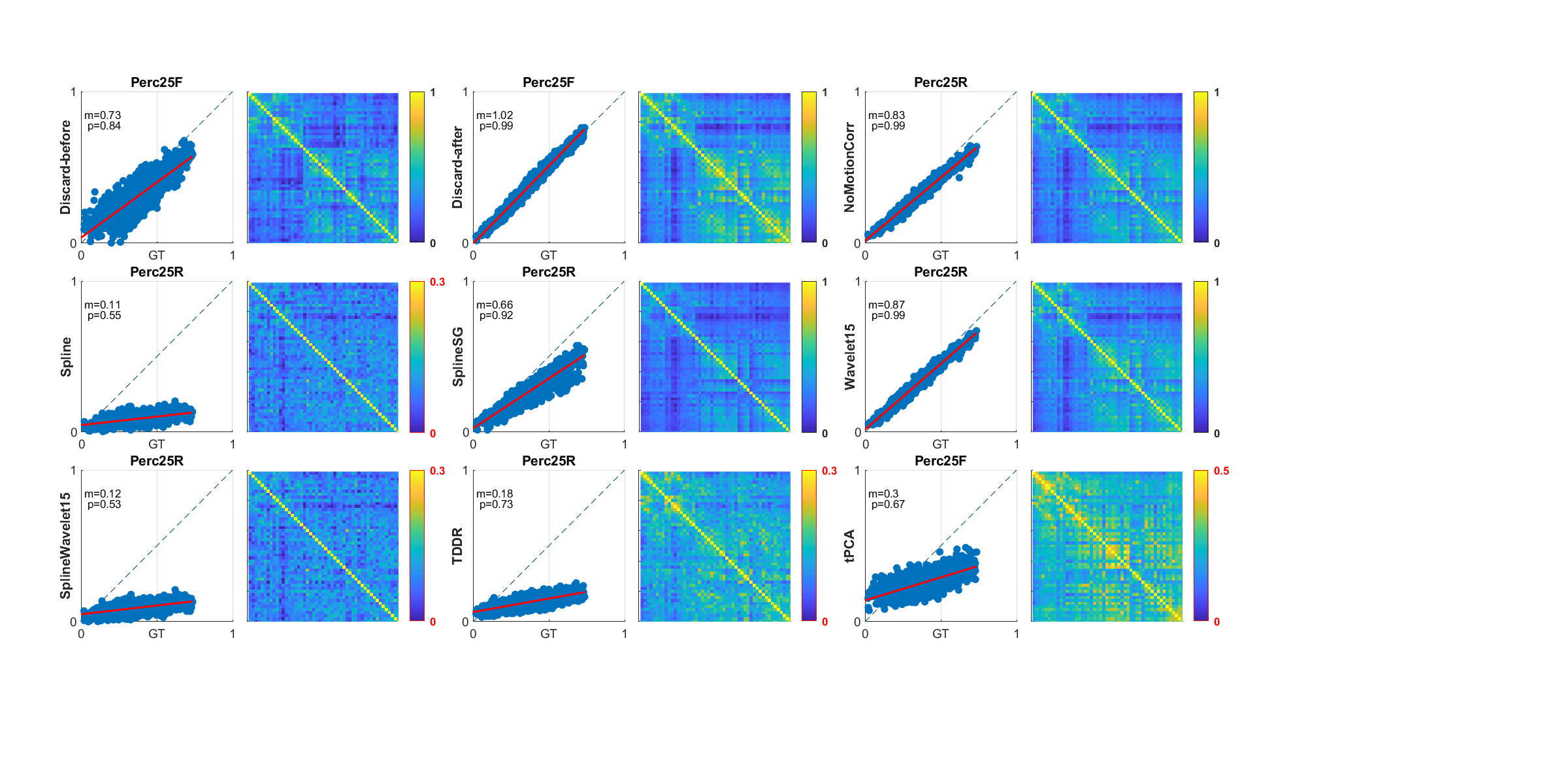


**Fig. S7** Scatterplots of testing and GT group correlation matrix in *Perc25* Only Spikes dataset and the correspondent group correlation matrix of the testing pipeline. The real color range is between zero and one. In specific cases, the range was reduced to highlight the testing correlation matrix pattern (please note the red values next to the color bars).


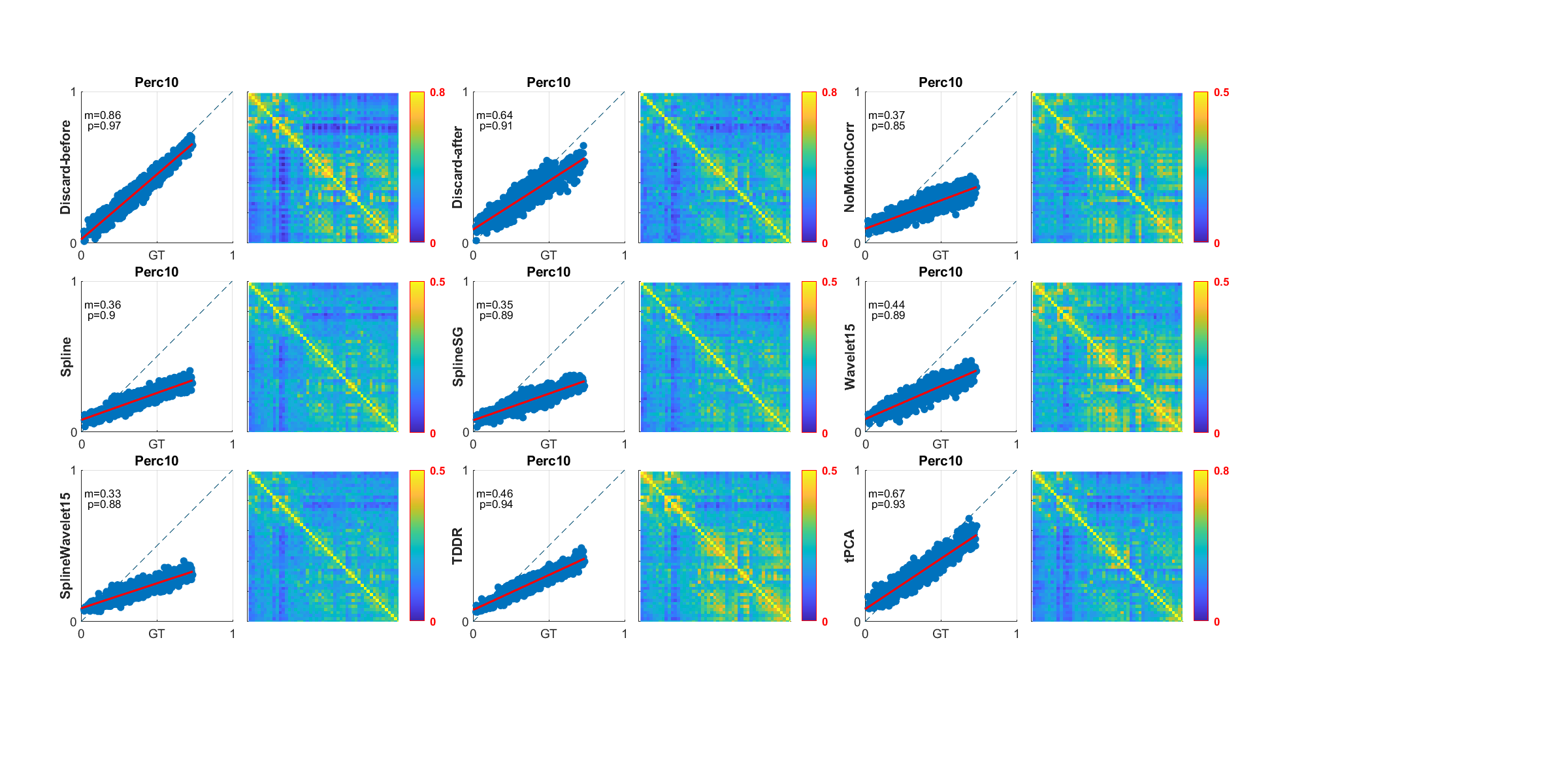


**Fig. S8** Scatterplots of testing and GT group correlation matrix in *Perc10* BS+Spikes dataset and the correspondent group correlation matrix of the testing pipeline. The real color range is between zero and one. In specific cases, the range was reduced to highlight the testing correlation matrix pattern (please note the red values next to the color bars).


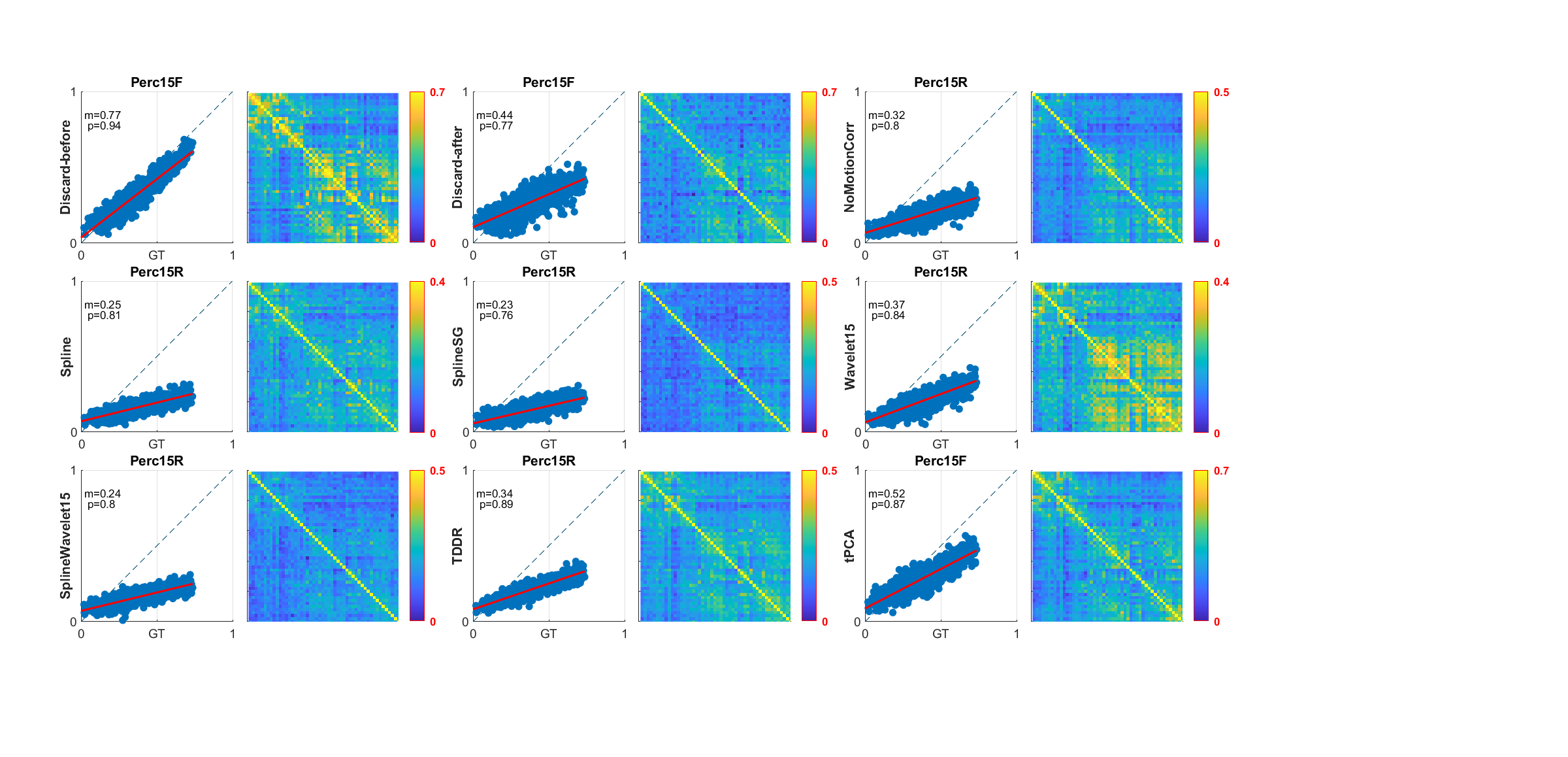


**Fig. S9** Scatterplots of testing and GT group correlation matrix in *Perc15* BS+Spikes dataset and the correspondent group correlation matrix of the testing pipeline. The real color range is between zero and one. In specific cases, the range was reduced to highlight the testing correlation matrix pattern (please note the red values next to the color bars).


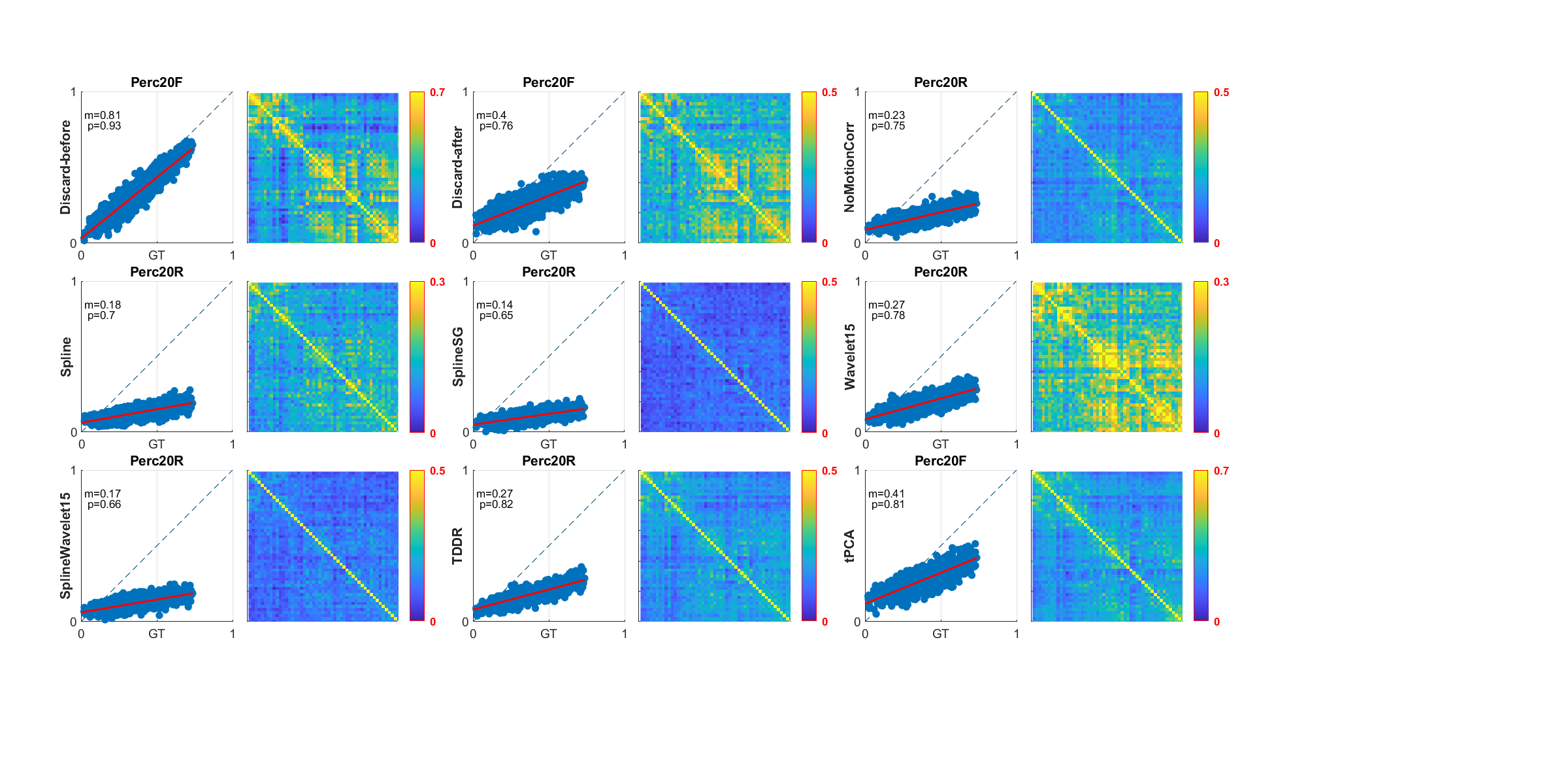


**Fig. S10** Scatterplots of testing and GT group correlation matrix in *Perc20* BS+Spikes dataset and the correspondent group correlation matrix of the testing pipeline. The real color range is between zero and one. In specific cases, the range was reduced to highlight the testing correlation matrix pattern (please note the red values next to the color bars).


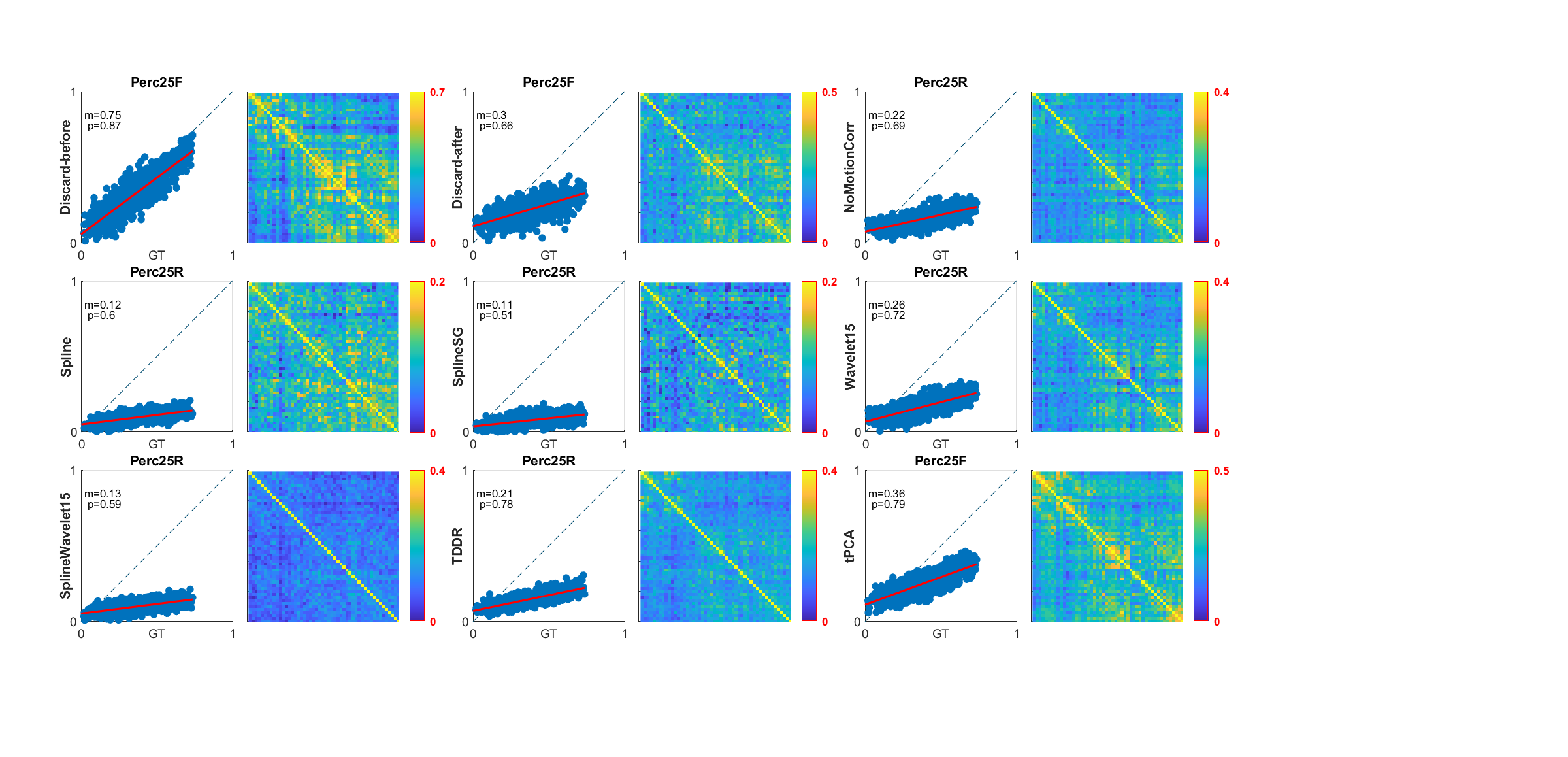


**Fig. S11** Scatterplots of testing and GT group correlation matrix in *Perc25* BS+Spikes dataset and the correspondent group correlation matrix of the testing pipeline. The real color range is between zero and one. In specific cases, the range was reduced to highlight the testing correlation matrix pattern (please note the red values next to the color bars).


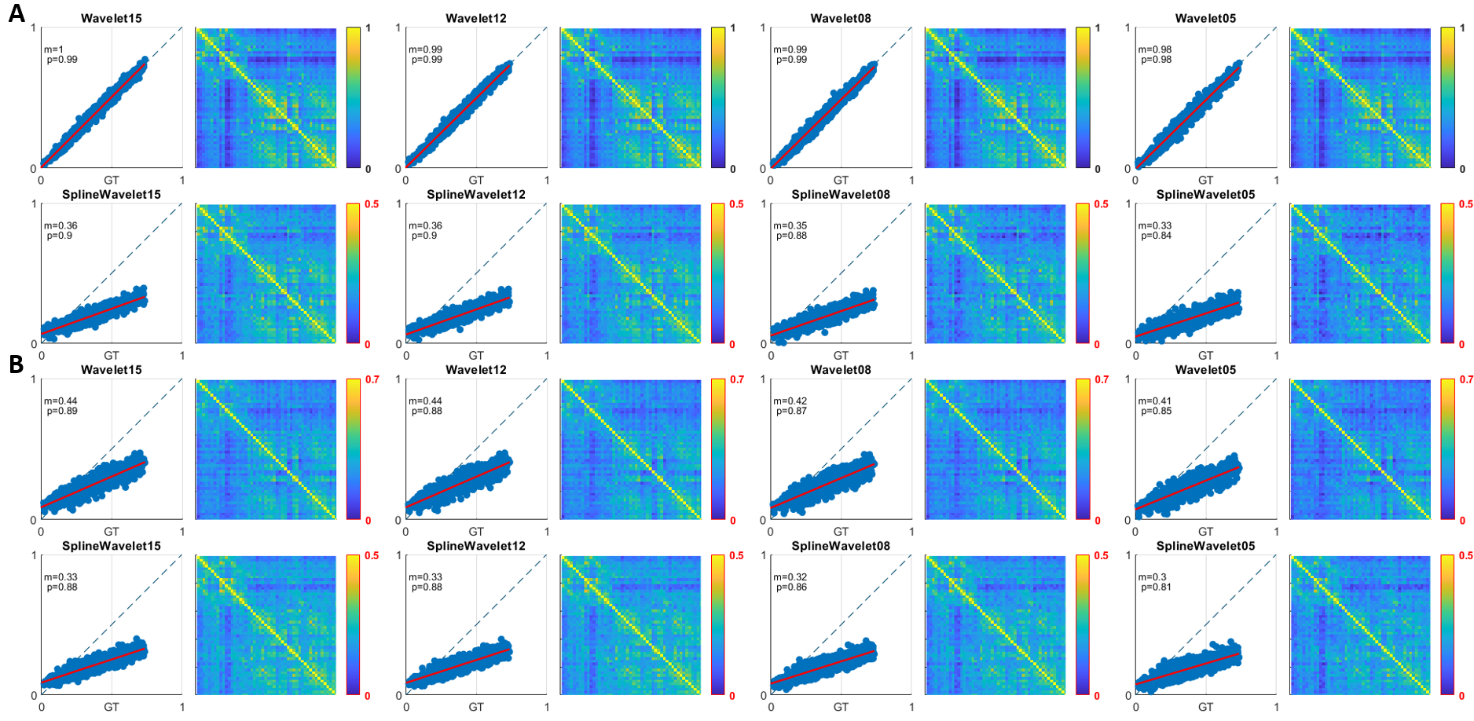
**Fig. S12** Scatterplots of testing and GT group correlation matrix in *Perc10* A) Only Spikes and B) BS+Spikes datasets and the correspondent group correlation matrix of the testing pipeline. The real color range is between zero and one. In specific cases, the range was reduced to highlight the testing correlation matrix pattern (please note the red values next to the color bars).


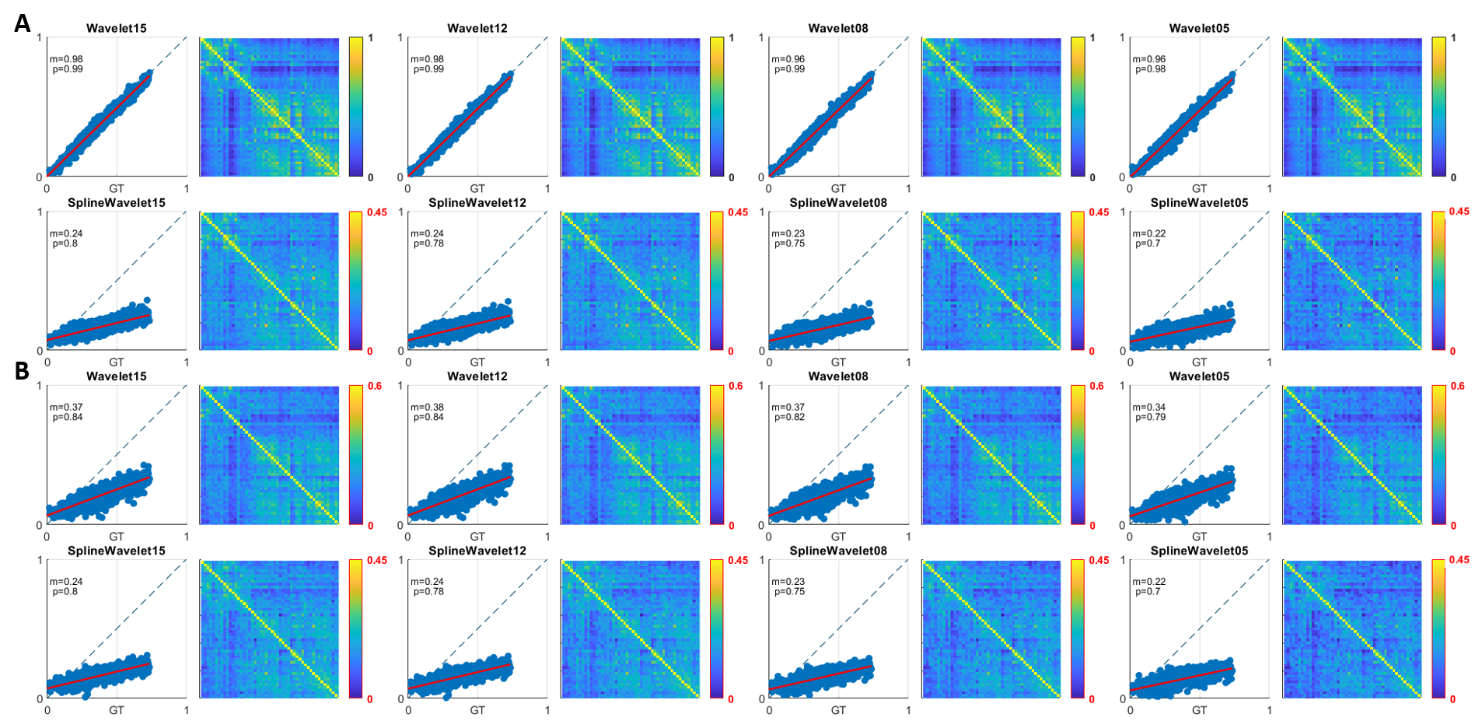


**Fig. S13** Scatterplots of testing and GT group correlation matrix in *Perc15* A) Only Spikes and B) BS+Spikes datasets and the correspondent group correlation matrix of the testing pipeline. The real color range is between zero and one. In specific cases, the range was reduced to highlight the testing correlation matrix pattern (please note the red values next to the color bars).


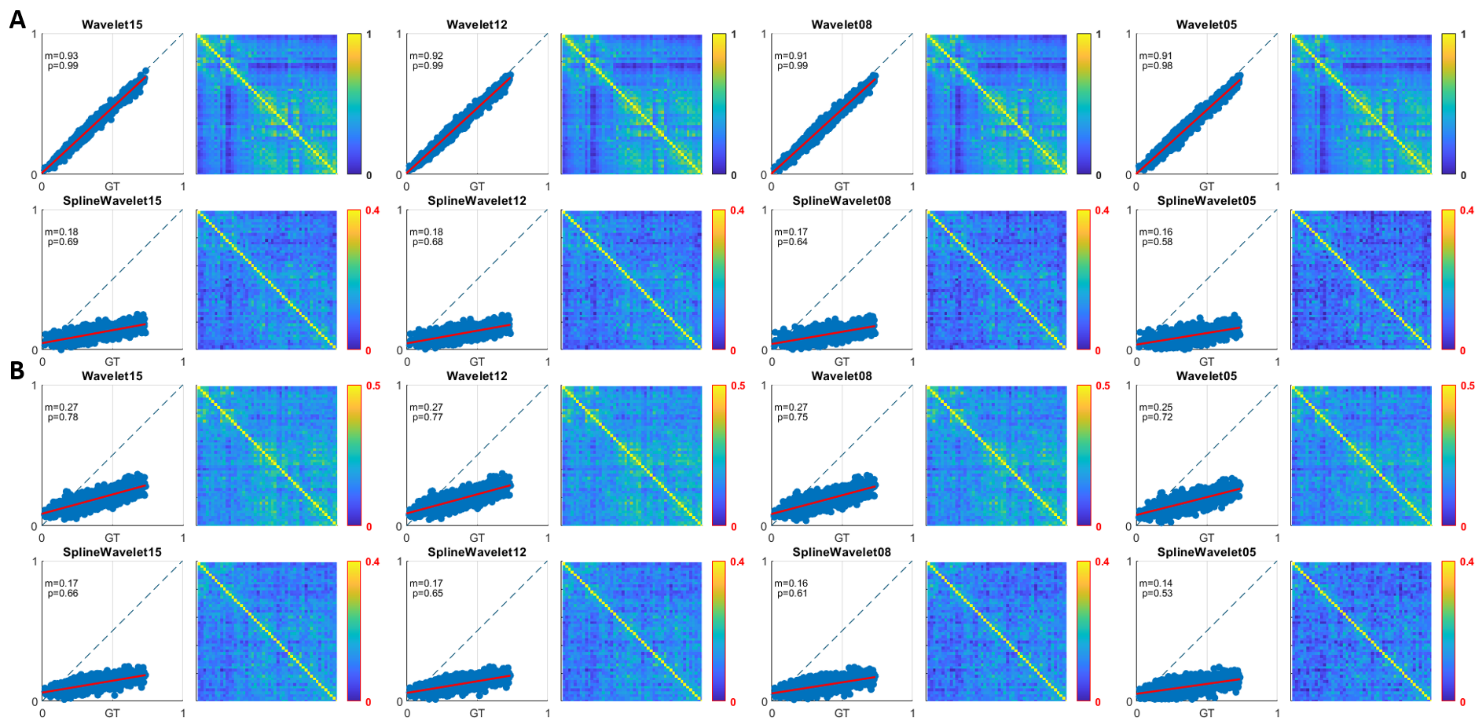


**Fig. S14** Scatterplots of testing and GT group correlation matrix in *Perc20* A) Only Spikes and B) BS+Spikes datasets and the correspondent group correlation matrix of the testing pipeline. The real color range is between zero and one. In specific cases, the range was reduced to highlight the testing correlation matrix pattern (please note the red values next to the color bars).


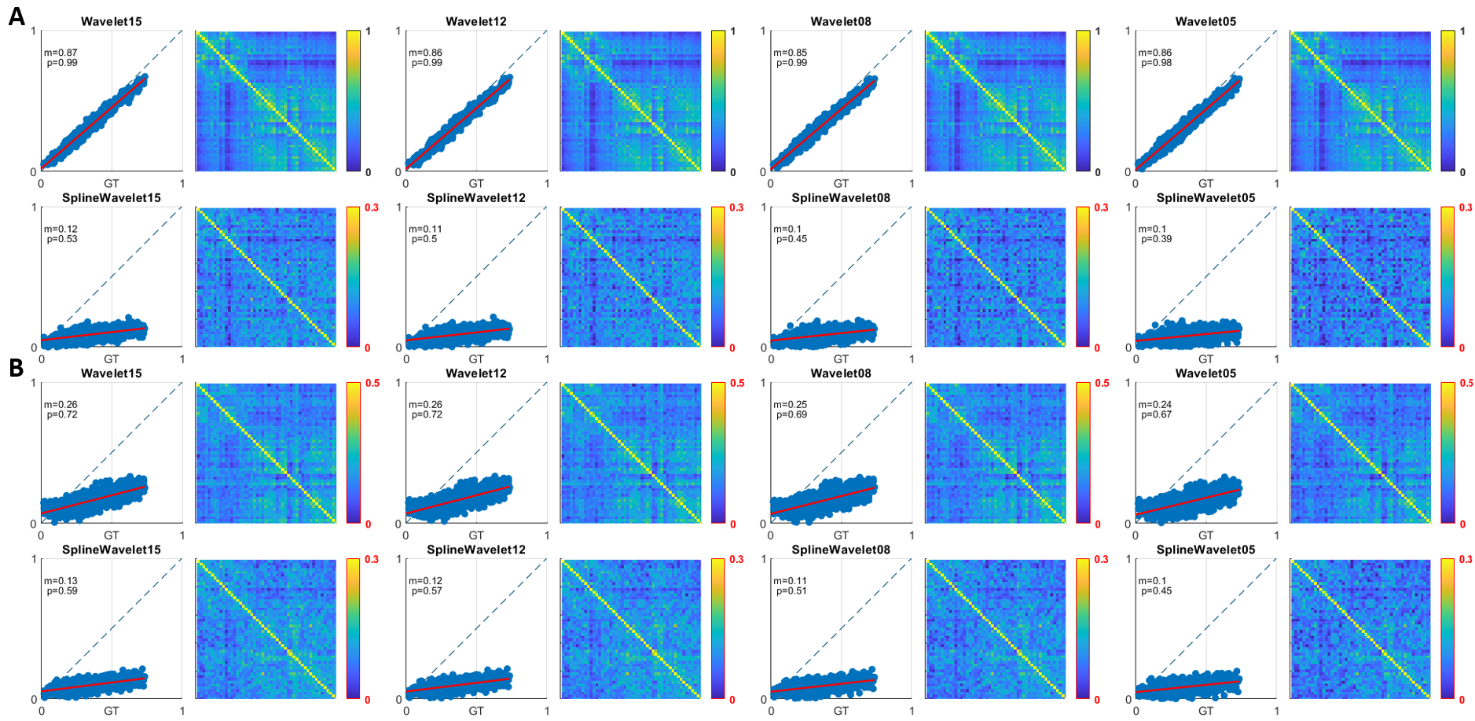


**Fig. S15** Scatterplots of testing and GT group correlation matrix in *Perc25* A) Only Spikes and B) BS+Spikes datasets and the correspondent group correlation matrix of the testing pipeline. The real color range is between zero and one. In specific cases, the range was reduced to highlight the testing correlation matrix pattern (please note the red values next to the color bars).

**Fig. S16** Optical Density signals before (blue lines) and after (orange lines) different motion correction techniques. Signals were randomly selected from a channel in A) Perc20 BS+Spikes Dataset and B) Perc20 Only Spikes datasets. Spline, Spline+Wavelet, SplineSG, Wavelet and TDDR motion correction techniques were applied on the Random dataset, while tPCA on the Fixed dataset.

**Table**

Table1: shows the statistical differences between methods for each dataset (Perc10, Perc15, Perc20, and Perc25 BS+Spike datasets).

|  | Perc10 | |  |  |  |  |  |  |  |  |  |  |  |  |  |
| --- | --- | --- | --- | --- | --- | --- | --- | --- | --- | --- | --- | --- | --- | --- | --- |
|  | Db | Da | NC | Sp | SpSG | W15 | W12 | W08 | W05 | SW15 | SW12 | SW08 | SW05 | TDDR | tPCA |
| Db |  | 0.27 | <.001 | <.001 | <.001 | <.001 | <.001 | <.001 | <.001 | <.001 | <.001 | <.001 | <.001 | <.001 | <.001 |
| Da |  |  | <.001 | <.001 | <.001 | <.001 | <.001 | <.001 | <.001 | <.001 | <.001 | <.001 | <.001 | <.001 | 1 |
| NC |  |  |  | 1 | <.001 | <.001 | <.001 | 0.01 | 1 | 1 | 0.41 | <.001 | <.001 | <.001 | <.001 |
| Sp |  |  |  |  | 1 | <.001 | <.001 | <.001 | 1 | 1 | 1 | 0.41 | <.001 | <.001 | <.001 |
| SpSG |  |  |  |  |  | <.001 | <.001 | <.001 | <.001 | 1 | 1 | 1 | <.001 | <.001 | <.001 |
| W15 |  |  |  |  |  |  | 1 | 1 | <.001 | <.001 | <.001 | <.001 | <.001 | 1 | <.001 |
| W12 |  |  |  |  |  |  |  | 1 | <.001 | <.001 | <.001 | <.001 | <.001 | 1 | <.001 |
| W08 |  |  |  |  |  |  |  |  | 0.09 | <.001 | <.001 | <.001 | <.001 | 1 | <.001 |
| W05 |  |  |  |  |  |  |  |  |  | 0.43 | 0.053 | <.001 | <.001 | <.001 | <.001 |
| SW15 |  |  |  |  |  |  |  |  |  |  | 1 | 1 | <.001 | <.001 | <.001 |
| SW12 |  |  |  |  |  |  |  |  |  |  |  | 1 | <.001 | <.001 | <.001 |
| SW08 |  |  |  |  |  |  |  |  |  |  |  |  | 0.83 | <.001 | <.001 |
| SW05 |  |  |  |  |  |  |  |  |  |  |  |  |  | <.001 | <.001 |
| TDDR |  |  |  |  |  |  |  |  |  |  |  |  |  |  | <.001 |
| tPCA |  |  |  |  |  |  |  |  |  |  |  |  |  |  |  |
|  |  |  |  |  |  |  |  |  |  |  |  |  |  |  |  |
|  | Perc15 | |  |  |  |  |  |  |  |  |  |  |  |  |  |
|  | Db | Da | NC | Sp | SpSG | W15 | W12 | W08 | W05 | SW15 | SW12 | SW08 | SW05 | TDDR | tPCA |
| Db |  | <.001 | <.001 | <.001 | <.001 | <.001 | <.001 | <.001 | <.001 | <.001 | <.001 | <.001 | <.001 | <.001 | <.001 |
| Da |  |  | <.001 | <.001 | <.001 | <.001 | <.001 | <.001 | <.001 | <.001 | <.001 | <.001 | <.001 | <.001 | 1 |
| NC |  |  |  | 1 | <.001 | <.001 | <.001 | <.001 | 1 | 0.57 | 0.08 | <.001 | <.001 | <.001 | <.001 |
| Sp |  |  |  |  | <.001 | <.001 | <.001 | <.001 | <.001 | 1 | 1 | 1 | <.001 | <.001 | <.001 |
| SpSG |  |  |  |  |  | <.001 | <.001 | <.001 | <.001 | <.001 | 0.18 | 1 | 1 | <.001 | <.001 |
| W15 |  |  |  |  |  |  | 1 | 1 | 0.46 | <.001 | <.001 | <.001 | <.001 | 1 | <.001 |
| W12 |  |  |  |  |  |  |  | 1 | 0.11 | <.001 | <.001 | <.001 | <.001 | 1 | <.001 |
| W08 |  |  |  |  |  |  |  |  | 1 | <.001 | <.001 | <.001 | <.001 | 1 | <.001 |
| W05 |  |  |  |  |  |  |  |  |  | <.001 | <.001 | <.001 | <.001 | 0.15 | <.001 |
| SW15 |  |  |  |  |  |  |  |  |  |  | 1 | 1 | <.001 | <.001 | <.001 |
| SW12 |  |  |  |  |  |  |  |  |  |  |  | 1 | 0.06 | <.001 | <.001 |
| SW08 |  |  |  |  |  |  |  |  |  |  |  |  | 1 | <.001 | <.001 |
| SW05 |  |  |  |  |  |  |  |  |  |  |  |  |  | <.001 | <.001 |
| TDDR |  |  |  |  |  |  |  |  |  |  |  |  |  |  | <.001 |
| tPCA |  |  |  |  |  |  |  |  |  |  |  |  |  |  |  |
|  |  |  |  |  |  |  |  |  |  |  |  |  |  |  |  |
|  | Perc20 | |  |  |  |  |  |  |  |  |  |  |  |  |  |
|  | Db | Da | NC | Sp | SpSG | W15 | W12 | W08 | W05 | SW15 | SW12 | SW08 | SW05 | TDDR | tPCA |
| Db |  | <.001 | <.001 | <.001 | <.001 | <.001 | <.001 | <.001 | <.001 | <.001 | <.001 | <.001 | <.001 | <.001 | <.001 |
| Da |  |  | <.001 | <.001 | <.001 | <.001 | <.001 | <.001 | <.001 | <.001 | <.001 | <.001 | <.001 | <.001 | 1 |
| NC |  |  |  | <.001 | <.001 | 0.12 | <.001 | 0.24 | 1 | <.001 | <.001 | <.001 | <.001 | 1 | <.001 |
| Sp |  |  |  |  | <.001 | <.001 | <.001 | <.001 | <.001 | 1 | 1 | 1 | <.001 | <.001 | <.001 |
| SpSG |  |  |  |  |  | <.001 | <.001 | <.001 | <.001 | <.001 | <.001 | 0.75 | 1 | <.001 | <.001 |
| W15 |  |  |  |  |  |  | 1 | 1 | 1 | <.001 | <.001 | <.001 | <.001 | 1 | <.001 |
| W12 |  |  |  |  |  |  |  | 1 | 1 | <.001 | <.001 | <.001 | <.001 | 1 | <.001 |
| W08 |  |  |  |  |  |  |  |  | 1 | <.001 | <.001 | <.001 | <.001 | 1 | <.001 |
| W05 |  |  |  |  |  |  |  |  |  | <.001 | <.001 | <.001 | <.001 | 1 | <.001 |
| SW15 |  |  |  |  |  |  |  |  |  |  | 1 | 1 | 0.18 | <.001 | <.001 |
| SW12 |  |  |  |  |  |  |  |  |  |  |  | 1 | 0.79 | <.001 | <.001 |
| SW08 |  |  |  |  |  |  |  |  |  |  |  |  | 1 | <.001 | <.001 |
| SW05 |  |  |  |  |  |  |  |  |  |  |  |  |  | <.001 | <.001 |
| TDDR |  |  |  |  |  |  |  |  |  |  |  |  |  |  | <.001 |
| tPCA |  |  |  |  |  |  |  |  |  |  |  |  |  |  |  |
|  |  |  |  |  |  |  |  |  |  |  |  |  |  |  |  |
|  | Perc25 | |  |  |  |  |  |  |  |  |  |  |  |  |  |
|  | Db | Da | NC | Sp | SpSG | W15 | W12 | W08 | W05 | SW15 | SW12 | SW08 | SW05 | TDDR | tPCA |
| Db |  | <.001 | <.001 | <.001 | <.001 | <.001 | <.001 | <.001 | <.001 | <.001 | <.001 | <.001 | <.001 | <.001 | <.001 |
| Da |  |  | <.001 | <.001 | <.001 | <.001 | <.001 | <.001 | <.001 | <.001 | <.001 | <.001 | <.001 | <.001 | 1 |
| NC |  |  |  | <.001 | <.001 | 1 | 1 | 1 | 1 | <.001 | <.001 | <.001 | <.001 | 1 | <.001 |
| Sp |  |  |  |  | <.001 | <.001 | <.001 | <.001 | <.001 | 1 | 1 | 1 | 1 | <.001 | <.001 |
| SpSG |  |  |  |  |  | <.001 | <.001 | <.001 | <.001 | <.001 | <.001 | 0.53 | 1 | <.001 | <.001 |
| W15 |  |  |  |  |  |  | 1 | 1 | 1 | <.001 | <.001 | <.001 | <.001 | 1 | <.001 |
| W12 |  |  |  |  |  |  |  | 1 | 1 | <.001 | <.001 | <.001 | <.001 | 1 | <.001 |
| W08 |  |  |  |  |  |  |  |  | 1 | <.001 | <.001 | <.001 | <.001 | 1 | <.001 |
| W05 |  |  |  |  |  |  |  |  |  | <.001 | <.001 | <.001 | <.001 | 1 | <.001 |
| SW15 |  |  |  |  |  |  |  |  |  |  | 1 | 1 | 1 | <.001 | <.001 |
| SW12 |  |  |  |  |  |  |  |  |  |  |  | 1 | 1 | <.001 | <.001 |
| SW08 |  |  |  |  |  |  |  |  |  |  |  |  | 1 | <.001 | <.001 |
| SW05 |  |  |  |  |  |  |  |  |  |  |  |  |  | <.001 | <.001 |
| TDDR |  |  |  |  |  |  |  |  |  |  |  |  |  |  | <.001 |
| tPCA |  |  |  |  |  |  |  |  |  |  |  |  |  |  |  |
|  |  |  |  |  |  |  |  |  |  |  |  |  |  |  |  |
